# Association of Weight‐Adjusted‐Waist Index With Brain Health: A 16‐Year Population‐Based Longitudinal Cohort Study

**DOI:** 10.1002/cns.71001

**Published:** 2026-06-23

**Authors:** Qi Sun, Li Yu, Ling Yang, Yanbo Liang, Qunya Qi, Ying Hui, Mingze Xu, Zhenchang Wang, Shouling Wu, Yuntao Wu, Han Lv

**Affiliations:** ^1^ Precision and Intelligence Medical Imaging Lab, Beijing Friendship Hospital Capital Medical University Beijing China; ^2^ Department of Radiology, Beijing Friendship Hospital Capital Medical University Beijing China; ^3^ Department of Health Data Science, School of Medical Technology Capital Medical University Beijing China; ^4^ Department of MRI Kailuan General Hospital Tangshan Hebei Province China; ^5^ Center for MRI Research, Academy for Advanced Interdisciplinary Studies Peking University Beijing China; ^6^ Department of Cardiology Kailuan General Hospital Tangshan Hebei Province China

**Keywords:** brain health, magnetic resonance imaging, obesity, weight‐adjusted‐waist index, white matter hyperintensity

## Abstract

**Background:**

The long‐term association of central obesity with brain structural integrity remains poorly understood. This study aimed to investigate the longitudinal association between cumulative Weight‐adjusted‐waist Index (WWI) exposure and multi‐modal neuroimaging markers of brain health.

**Methods:**

This prospective community‐based cohort study included 935 participants from the META‐KLS Study. Cumulative WWI was calculated as the time‐weighted average over 12 years prior to MRI acquisition. Neuroimaging outcomes included regional gray matter volume, white matter hyperintensity (WMH), and diffusion tensor imaging (DTI) metrics. Generalized linear models, restricted cubic splines, and mediation analyzes were performed.

**Results:**

Elevated cumulative WWI was associated with adverse brain structural outcomes, particularly in females. In women, higher WWI was linked to extensive WMH burden (*p*_FDR_ = 0.002), widespread microstructural disintegration (*p*_FDR_ = 0.023), and specific atrophy in the orbital frontal cortex. A J‐shaped dose–response relationship was identified for white matter injury, suggesting a tipping point for metabolic resilience. In exploratory mediation analyzes, FBG, SBP, and hs‐CRP statistically accounted for 14.7%, 11.3%, and 11.3% of the association between cumulative WWI and WMH burden, respectively, while SBP accounted for 17.8% of the association with global MD.

**Conclusion:**

Cumulative WWI serves as a potential predictor of adverse brain structural outcomes, particularly manifesting as white matter injury and atrophy in women. Early monitoring of WWI offers a vital window for targeted metabolic interventions to preserve brain structural integrity.

## Graphical abstract

Cumulative central obesity, assessed by the Weight‐adjusted‐waist Index (WWI), is associated with adverse brain structural outcomes over a 16‐year study period, particularly in females. These structural associations primarily involve white matter injury and frontal atrophy, and are potentially mediated by metabolic inflammation and hemodynamic stress.

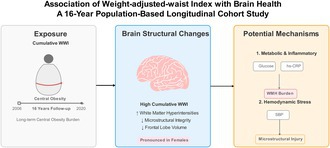

## 1 Introduction

Brain health is defined as the preservation of optimal brain integrity and cognitive function in the absence of overt neurological disorders [1]. It can be influenced by a wide range of conditions, particularly obesity [2, 3]. Magnetic resonance imaging (MRI)‐based neuroimaging features allow for the objective assessment of brain health [4, 5].

Over the past two decades, obesity has progressively emerged as a substantial contributor to the global burden of disease [6, 7]. Central obesity, characterized by excessive abdominal adipose tissue accumulation, is a risk factor for poorer brain health, accelerating cognitive decline and increasing dementia incidence [8]. While body mass index (BMI) has traditionally been the standard measure for obesity, its limitations are increasingly recognized; BMI fails to distinguish between fat mass and muscle mass [9].

While waist circumference (WC) primarily reflects central adiposity, it is heavily confounded by body size. In contrast, the Weight‐adjusted‐waist Index (WWI) standardizes WC by weight, theoretically making it an anthropometric index that may reflect adverse body composition characterized by relatively greater central adiposity in relation to body weight [10, 11, 12]. Several cross‐sectional studies that acquired WWI and cognitive data simultaneously found that individuals with high WWI had poorer cognitive performance [13, 14, 15, 16], but the association with specific brain structural markers remains to be elucidated.

Given that central obesity represents a chronic condition characterized by long‐term development, a cumulative burden of WWI, rather than a cross‐sectional value of WWI, is more rational to reflect the burden of central obesity [17]. However, the association between cumulative WWI exposure and brain health remains unclear. Existing evidence has primarily focused on cognitive function outcomes, with limited exploration of structural brain changes. Furthermore, potential sex‐specific differences in the association between central obesity and brain health are unclear [18]. A reference value of WWI would be helpful in promoting brain health. More importantly, WWI and brain health may be associated with various factors in the real world. The role of systemic inflammation in mediating the relationship between cumulative WWI and brain health, as well as potential sex‐specific differences, warrants further investigation [19].

In this study, it was hypothesized that elevated cumulative WWI exposure is negatively associated with brain health. Therefore, this study aimed to investigate the association between cumulative WWI and various neuroimaging markers. Furthermore, potential sex‐specific vulnerabilities and the potential mediating roles of metabolic and inflammatory factors were explored. The findings may offer novel insights for brain health preservation strategies.

## 2 Methods

### 2.1 Design and Participants

This study uses data from the Multimodality Medical Imaging Study Based on Kailuan Study (META‐KLS), an ongoing neuroimaging initiative nested within the prospective Kailuan Study. Established in 2006 in Tangshan, China, the parent Kailuan cohort includes employees of the Kailuan Group and community residents. The META‐KLS sub‐study, launched in 2020, specifically recruits participants for advanced neuroimaging evaluations. Methodological details of the META‐KLS have been reported elsewhere [20]. The protocol for this research received formal approval from the Medical Ethics Committee of Kailuan General Hospital (No: 2021002). Prior to their inclusion, all participants provided written informed consent. No financial stipend was offered for participation.

Participants in the META‐KLS were initially enrolled in this study (*N* = 1181). A total of 145 participants with missing cumulative WWI data were excluded. Subsequently, 101 participants were excluded due to missing MRI data or data with poor quality. The final analytical sample included 935 participants. The study flowchart is presented in Figure S1. To address potential selection bias due to loss to follow‐up or missing data over the 12‐year period, we compared the baseline characteristics of the included analytic sample (*N* = 935) with those of the excluded participants (*N* = 246) due to missing cumulative WWI or MRI data (Table S1). The two groups were well‐matched in terms of sex, education level, alcohol consumption, and baseline systolic blood pressure (all *p* > 0.05). Excluded participants were slightly younger on average (51.6 vs. 56.0 years, *p* < 0.001) and had a marginally higher proportion of ever‐smokers. This age difference is largely attributed to younger, working‐age participants having higher attrition rates over the long‐term follow‐up due to busy schedules, making them more likely to miss the time‐consuming MRI scan.

### 2.2 Measurements of Clinical Features

Demographic and clinical data were obtained through biennial face‐to‐face interviews and physical examinations conducted from 2006 to 2020. Baseline covariates (assessed in 2006) included sex (male or female), educational attainment (low, middle school or below; high, high school or above), smoking and drinking habits (never or ever), baseline physical activity (categorized as none, occasional, or frequent exercise), and systolic blood pressure (SBP, mmHg). Age (years) was recorded at the time of MRI acquisition. Laboratory analysis of overnight fasting blood samples determined concentrations of fasting blood glucose (FBG, mmol/L), lipids (triglycerides, LDL‐C, and HDL‐C; all in mmol/L), and high‐sensitivity C‐reactive protein (hs‐CRP). For mediation analysis, metabolic and inflammatory markers were assessed at the 2020 examination, concurrent with the neuroimaging assessment.

### 2.3 Cumulative WWI Exposure

WWI was calculated as WC (cm) divided by the square root of body weight (kg) [12]. To capture long‐term exposure, cumulative WWI was derived using data from five biennial examinations spanning 2006 to 2018, prior to the neuroimaging assessment. The cumulative burden was quantified as the time‐weighted average WWI, computed via the trapezoidal rule by dividing the total area under the WWI‐time curve by the total follow‐up duration [21].

### 2.4 Neuroimaging Data Acquisition

Brain MRI scanning was performed on a 3.0T GE MR750 system (Milwaukee, WI, USA) at Kailuan General Hospital between 2020 and 2022. The imaging protocol included high‐resolution 3D T1‐weighted imaging (3D‐BRAVO) for volumetric analysis, 3D Fluid‐Attenuated Inversion Recovery (FLAIR) for white matter hyperintensity (WMH) assessment, and Diffusion Tensor Imaging (DTI) for microstructural integrity.

Details of the neuroimaging acquisition parameters were as follows: T1‐weighted images were acquired using a 3D Fast Spoiled Gradient Echo (FSPGR) sequence (TR = 6.6 ms, TE = 2.9 ms, FOV = 256 × 256 mm, Matrix = 256 × 256, Slice Thickness = 1.0 mm); FLAIR images were acquired with TR = 9500 ms, TE = 120 ms, Inversion Time = 2250 ms; DTI was performed using a single‐shot spin‐echo Echo‐Planar Imaging sequence (TR = 11,000 ms, TE = 98 ms, 30 directions, b = 1000 s/mm^2^).

### 2.5 Neuroimaging Data Processing

A standardized pipeline was employed for image processing. For macrostructural analysis, total intracranial volume (TIV) and gray matter (GM) volume were automatically segmented using Statistical Parametric Mapping (SPM12). Regional GM volumes were extracted based on the AAL 90 atlas.

WMH burden, which included total periventricular (PWMH) and deep (DWMH) lesions, was quantified from FLAIR images. To correct for head size differences, WMH volumes were normalized to TIV and expressed as percentages. Microstructural integrity was assessed using DTI‐derived fractional anisotropy (FA) and mean diffusivity (MD). Parametric maps were generated, and mean values were extracted for 48 white matter tracts defined by the JHU atlas.

### 2.6 Statistical Analyzes

Continuous variables with normal distribution were presented as mean (standard deviation [SD]), while those with skewed distribution were reported as median (interquartile range [IQR]). Categorical variables were expressed as frequencies and percentages. Differences between WWI tertile groups were compared using one‐way analysis of variance (ANOVA) for normally distributed continuous variables, Kruskal‐Wallis test for skewed continuous variables, and Chi‐square test for categorical variables. Missing baseline covariates within the analytic sample were imputed using chained equations before model fitting to minimize additional loss of observations due to covariate missingness.

Generalized linear models were used to evaluate the association between cumulative WWI (standardized) and neuroimaging features. WWI values were categorized into tertiles, using the category with the lowest WWI as the reference. Model 1 was pre‐specified as a minimally adjusted model including age and sex. Model 2 was pre‐specified as the fully adjusted model, additionally including education level, smoking status, drinking status, physical activity, systolic blood pressure, diabetes status, LDL‐C, HDL‐C, triglycerides, and history of hypertension. This stepwise strategy was used to evaluate whether the associations were robust to progressive adjustment for demographic, lifestyle, and vascular‐metabolic factors. Subgroup analyzes stratified by sex and age at MRI (< 60 vs. ≥ 60 years) were performed to explore potential specific associations. The statistical significance of the sex‐by‐WWI interaction was evaluated by comparing models with and without the interaction term using ANOVA.

Regional analyzes were conducted to assess the association between cumulative WWI and specific brain structures, including regional gray matter volumes (AAL90 atlas), regional WMH burden (lobar parcellation), and tract‐specific DTI metrics (JHU atlas). For the regional analyzes, to avoid over‐adjustment bias and to follow standard neuroimaging protocols, the models were adjusted for age, sex, and TIV. To account for multiple comparisons, *p*‐values were corrected using the Benjamini‐Hochberg false discovery rate (FDR) procedure. For the primary, subgroup, and sensitivity analyzes, FDR correction was applied separately within each model across the 10 predefined neuroimaging outcomes, rather than across all analyzes in the entire study. For the regional exploratory analyzes, FDR correction was independently applied across regions within each specific atlas or imaging domain, including the AAL90 gray matter atlas, lobar WMH parcellation, and JHU white matter tract atlas.

Restricted cubic splines (RCS) with three knots (at the 10th, 50th, and 90th percentiles) were employed to detect potential non‐linear relationships. Exploratory statistical mediation analyzes were conducted using the mediation R package with 1000 simulations to examine whether FBG, SBP, and hs‐CRP statistically accounted for part of the associations between cumulative WWI and neuroimaging outcomes. Because these candidate mediators and neuroimaging outcomes were assessed concurrently at the MRI phase, these analyzes were not interpreted as definitive causal mediation and were considered hypothesis‐generating.

Sensitivity analyzes were performed to examine the stability of the main findings under alternative model specifications and sample restrictions. First, BMI was additionally included in the fully adjusted model as an exploratory sensitivity analysis to assess whether the associations were directionally consistent after accounting for general adiposity; given the mathematical overlap between WWI and BMI through body weight, these BMI‐adjusted results were interpreted cautiously. Second, the main analyzes were repeated after excluding participants with hypertension or diabetes. Third, the analyzes were repeated after excluding participants with a history of cardiovascular disease. All statistical analyzes were performed using R software (version 4.3.3).

## 3 Results

### 3.1 Baseline Characteristics of Participants

A total of 935 participants were eligible for this study. Table 1 lists the participants' clinical characteristics according to cumulative WWI tertiles. Compared with participants in the low WWI group, those in the high WWI group were significantly older, more likely to be male, and had a lower education level. Metabolically, higher cumulative WWI was associated with an adverse profile, including higher SBP, higher triglycerides, higher FBG, and a higher prevalence of hypertension.

**TABLE 1:** Baseline characteristics of study participants.

Characteristic	WWI			*p*
	Low (*n* = 319)	Medium (*n* = 308)	High (*n* = 308)	
No. of participants	319	308	308	
Age, years, Median [IQR]	51.00 [44.00, 58.00]	58.00 [50.00, 66.00]	61.00 [53.00, 68.00]	< 0.001
Sex				< 0.001
Female	194 (60.8)	127 (41.2)	117 (38.0)	
Male	125 (39.2)	181 (58.8)	191 (62.0)	
Education level				< 0.001
Low	98 (30.7)	149 (48.4)	158 (51.3)	
High	221 (69.3)	159 (51.6)	150 (48.7)	
Smoking status				< 0.001
Never	260 (81.5)	230 (74.7)	195 (63.3)	
Ever	59 (18.5)	78 (25.3)	113 (36.7)	
Drinking status				< 0.001
Never	214 (67.1)	182 (59.1)	151 (49.0)	
Ever	105 (32.9)	126 (40.9)	157 (51.0)	
SBP, mmHg, Median [IQR]	110.00 [100.70, 120.70]	119.32 [109.30, 129.30]	120.00 [109.30, 130.00]	< 0.001
FBG, mmol/L, Median [IQR]	4.96 [4.53, 5.27]	5.01 [4.66, 5.42]	5.06 [4.57, 5.51]	0.006
LDL‐C, mmol/L, Median [IQR]	2.10 [1.66, 2.54]	2.16 [1.80, 2.70]	2.17 [1.71, 2.64]	0.145
HDL‐C, mmol/L, Median [IQR]	1.50 [1.29, 1.71]	1.47 [1.24, 1.65]	1.49 [1.26, 1.72]	0.305
Triglycerides, mmol/L, Median [IQR]	0.97 [0.67, 1.51]	1.19 [0.82, 1.77]	1.35 [0.88, 2.07]	< 0.001
History of hypertension				0.001
No	302 (94.7)	282 (91.6)	266 (86.4)	
Yes	17 (5.3)	26 (8.4)	42 (13.6)	
History of hyperlipidemia				0.926
No	313 (98.1)	301 (97.7)	301 (97.7)	
Yes	6 (1.9)	7 (2.3)	7 (2.3)	
Diabetes				0.061
No	310 (97.2)	294 (95.5)	287 (93.2)	
Yes	9 (2.8)	14 (4.5)	21 (6.8)	
History of CVD				0.06
No	319 (100.0)	306 (99.4)	303 (98.4)	
Yes	0 (0.0)	2 (0.6)	5 (1.6)	

*Note:* Data are presented as Median [IQR] for non‐normally distributed continuous variables (Age, Systolic Blood Pressure, Fasting Blood Glucose, LDL‐C, HDL‐C, and Triglycerides) and *N* (%) for categorical variables. *p* values were calculated using the Kruskal‐Wallis test for continuous variables and the Chi‐square test for categorical variables.

Abbreviations: FBG, fasting blood glucose; SBP, systolic blood pressure.

### 3.2 Association of Cumulative WWI With Neuroimaging Markers

After adjustment for confounders and applying FDR correction, compared with participants in the lowest tertile of cumulative WWI, those in the highest tertile exhibited significantly smaller brain parenchyma volume (β = −0.794; 95% CI, −1.254 to −0.333; *p*_FDR_ = 0.003) and white matter volume (β = −0.556; 95% CI, −0.849 to −0.263; *p*_FDR_ = 0.001). Conversely, high cumulative WWI was associated with greater CSF volume (β = 0.757; 95% CI, 0.304 to 1.211; *p*_FDR_ = 0.004). High cumulative WWI was significantly associated with a greater burden of total WMH (β = 0.230; 95% CI, 0.109 to 0.352; *p*_FDR_ = 0.001), periventricular WMH (β = 0.104; 95% CI, 0.053 to 0.155; *p*_FDR_ < 0.001), and deep WMH (β = 0.126; 95% CI, 0.047 to 0.204; *p*_FDR_ = 0.005). Additionally, high cumulative WWI was linked to increased global MD (β = 0.257; 95% CI, 0.128 to 0.385; *p*_FDR_ < 0.001). No significant associations were observed between cumulative WWI and gray matter volume, hippocampus volume, or global FA (Table 2). In the exploratory BMI‐adjusted sensitivity analysis, the associations with brain volume metrics were attenuated, whereas the associations with white matter injury remained directionally consistent but were weakened after FDR correction (Table S2).

**TABLE 2:** Association between cumulative WWI and neuroimaging markers in the total population.

Neuroimaging features	Models	WWI				*p*_FDR_
		Low	Medium beta (95% CI)	High beta (95% CI)	*p* for trend	
Brain parenchyma volume	Model 1	Ref	−0.047 (−0.495, 0.401)	−0.868 (−1.328, −0.408)	< 0.001	< 0.001
	Model 2	Ref	0.030 (−0.418, 0.477)	−0.794 (−1.254, −0.333)	0.001	0.003
Gray matter volume	Model 1	Ref	−0.038 (−0.343, 0.267)	−0.292 (−0.605, 0.021)	0.066	0.151
	Model 2	Ref	0.009 (−0.297, 0.314)	−0.237 (−0.552, 0.077)	0.136	0.309
White matter volume	Model 1	Ref	−0.009 (−0.293, 0.274)	−0.576 (−0.867, −0.285)	< 0.001	< 0.001
	Model 2	Ref	0.021 (−0.263, 0.305)	−0.556 (−0.849, −0.263)	< 0.001	0.001
CSF volume	Model 1	Ref	0.055 (−0.386, 0.496)	0.829 (0.377, 1.281)	< 0.001	< 0.001
	Model 2	Ref	−0.020 (−0.460, 0.420)	0.757 (0.304, 1.211)	0.001	0.004
Hippocampus volume	Model 1	Ref	0.001 (−0.003, 0.004)	−0.004 (−0.007, −0.001)	0.02	0.054
	Model 2	Ref	0.001 (−0.002, 0.004)	−0.004 (−0.007, −0.000)	0.031	0.08
Total WMH volume	Model 1	Ref	−0.041 (−0.162, 0.081)	0.269 (0.144, 0.393)	< 0.001	< 0.001
	Model 2	Ref	−0.065 (−0.184, 0.053)	0.230 (0.109, 0.352)	< 0.001	0.001
Periventricular WMH	Model 1	Ref	−0.007 (−0.057, 0.044)	0.120 (0.068, 0.171)	< 0.001	< 0.001
	Model 2	Ref	−0.017 (−0.066, 0.033)	0.104 (0.053, 0.155)	< 0.001	< 0.001
Deep WMH	Model 1	Ref	−0.034 (−0.112, 0.044)	0.148 (0.068, 0.229)	< 0.001	< 0.001
	Model 2	Ref	−0.049 (−0.125, 0.028)	0.126 (0.047, 0.204)	0.002	0.005
Global FA	Model 1	Ref	0.026 (−0.118, 0.169)	−0.081 (−0.229, 0.066)	0.275	0.562
	Model 2	Ref	0.028 (−0.116, 0.172)	−0.075 (−0.224, 0.073)	0.315	0.534
Global MD	Model 1	Ref	0.019 (−0.106, 0.143)	0.276 (0.148, 0.405)	< 0.001	< 0.001
	Model 2	Ref	0.005 (−0.119, 0.130)	0.257 (0.128, 0.385)	< 0.001	< 0.001

*Note:* Model 1 was pre‐specified as a minimally adjusted model including age and sex. Model 2 was pre‐specified as the fully adjusted model including educational level, smoking status, drinking status, physical activity, systolic blood pressure, diabetes status, LDL‐C, HDL‐C, triglycerides, and history of hypertension. *p*_FDR_ represents the FDR‐corrected *p*‐value for the highest WWI tertile compared with the lowest tertile, adjusted across the 10 predefined neuroimaging outcomes within each model.

Abbreviations: CI, confidence interval; FA, fractional anisotropy; FDR, false discovery rate; MD, mean diffusivity; Ref, reference; WMH, white matter hyperintensity; WWI, weight‐adjusted‐waist index.

### 3.3 Sex‐ and Age‐Stratified Subgroup Analyzes

Regarding the results stratified by sex, the associations of high cumulative WWI and neuroimaging features were generally more pronounced in females. In females, high cumulative WWI (highest versus lowest tertile) was significantly associated with reduced brain parenchyma volume (*p*_FDR_ = 0.005) and white matter volume (*p*_FDR_ = 0.007), and increased CSF volume (*p*_FDR_ = 0.005). Furthermore, females exhibited significantly increased burdens of Total WMH, PWMH, and DWMH (all *p*_FDR_ ≤ 0.003), as well as increased Global MD (*p*_FDR_ = 0.023). In males, although nominal associations were observed for white matter volume and WMH, none remained statistically significant after stringent FDR correction. A significant sex interaction was observed for global MD (*p* for interaction = 0.011), indicating a stronger association in females (Table 3).

**TABLE 3:** Association between cumulative WWI and neuroimaging markers stratified by sex.

Outcome	WWI	Male		Female		*p* for interaction
		β (95% CI)	*p* (*p*_FDR_)	β (95% CI)	*p* (*p*_FDR_)	
Brain parenchyma volume	Low	Ref.		Ref.		0.702
	Medium	0.052 (−0.586, 0.689)	0.874 (0.920)	−0.054 (−0.700, 0.591)	0.869 (0.910)	
	High	−0.623 (−1.283, 0.037)	0.065 (0.186)	−1.101 (−1.758, −0.443)	0.001 (0.005)	
Gray matter volume	Low	Ref.		Ref.		0.568
	Medium	0.091 (−0.322, 0.503)	0.667 (0.833)	−0.130 (−0.598, 0.337)	0.585 (0.836)	
	High	−0.103 (−0.530, 0.323)	0.635 (0.833)	−0.456 (−0.933, 0.020)	0.061 (0.136)	
White matter volume	Low	Ref.		Ref.		0.702
	Medium	−0.039 (−0.448, 0.370)	0.852 (0.920)	0.076 (−0.327, 0.479)	0.711 (0.910)	
	High	−0.520 (−0.943, −0.096)	0.017 (0.083)	−0.644 (−1.055, −0.234)	0.002 (0.007)	
CSF volume	Low	Ref.		Ref.		0.609
	Medium	−0.024 (−0.648, 0.600)	0.939 (0.939)	0.037 (−0.604, 0.678)	0.910 (0.910)	
	High	0.581 (−0.064, 1.227)	0.078 (0.195)	1.072 (0.420, 1.725)	0.001 (0.005)	
Hippocampus volume	Low	Ref.		Ref.		0.085
	Medium	−0.001 (−0.005, 0.004)	0.784 (0.920)	0.001 (−0.004, 0.005)	0.771 (0.910)	
	High	−0.006 (−0.011, −0.001)	0.011 (0.076)	−0.002 (−0.007, 0.003)	0.427 (0.712)	
Total WMH volume	Low	Ref.		Ref.		0.092
	Medium	−0.112 (−0.319, 0.094)	0.288 (0.564)	0.032 (−0.068, 0.132)	0.527 (0.811)	
	High	0.250 (0.037, 0.464)	0.022 (0.088)	0.202 (0.100, 0.303)	< 0.001 (0.002)	
Periventricular WMH	Low	Ref.		Ref.		0.064
	Medium	−0.037 (−0.120, 0.047)	0.390 (0.650)	0.025 (−0.022, 0.073)	0.300 (0.559)	
	High	0.114 (0.028, 0.201)	0.010 (0.076)	0.087 (0.039, 0.135)	< 0.001 (0.003)	
Deep WMH	Low	Ref.		Ref.		0.176
	Medium	−0.075 (−0.210, 0.060)	0.275 (0.564)	0.007 (−0.055, 0.069)	0.824 (0.910)	
	High	0.135 (−0.004, 0.275)	0.057 (0.186)	0.114 (0.051, 0.177)	< 0.001 (0.003)	
Global FA	Low	Ref.		Ref.		0.087
	Medium	0.074 (−0.172, 0.320)	0.557 (0.796)	−0.072 (−0.210, 0.066)	0.308 (0.559)	
	High	−0.132 (−0.388, 0.123)	0.310 (0.564)	−0.014 (−0.155, 0.127)	0.847 (0.910)	
Global MD	Low	Ref.		Ref.		0.011
	Medium	−0.075 (−0.276, 0.127)	0.467 (0.718)	0.143 (0.002, 0.283)	0.047 (0.118)	
	High	0.306 (0.097, 0.515)	0.002 (0.076)	0.195 (0.051, 0.339)	0.008 (0.023)	

*Note:* The models were adjusted for age, educational level, smoking status, drinking status, physical activity, systolic blood pressure, diabetes status, LDL‐C, HDL‐C, triglycerides, and history of hypertension. *p* represents the raw *p*‐value, and PFDR represents the FDR‐corrected *p*‐value adjusted across the 10 predefined neuroimaging outcomes separately within each sex‐specific model.

Abbreviations: CI, confidence interval; CSF, cerebrospinal fluid; FA, fractional anisotropy; MD, mean diffusivity; Ref, reference; WMH, white matter hyperintensity; WWI, weight‐adjusted‐waist index.

To further validate the stability of our findings and address the dominant role of aging in brain structural alterations, we performed subgroup analyzes stratified by age at MRI (< 60 vs. ≥ 60 years) (Table S3). Interestingly, the adverse association of cumulative WWI on brain structural markers was substantially more pronounced in the midlife stratum (< 60 years, *p* for trend < 0.05), while the associations became attenuated in the older stratum (≥ 60 years).

### 3.4 Dose–Response Relationships

**FIGURE 1:**
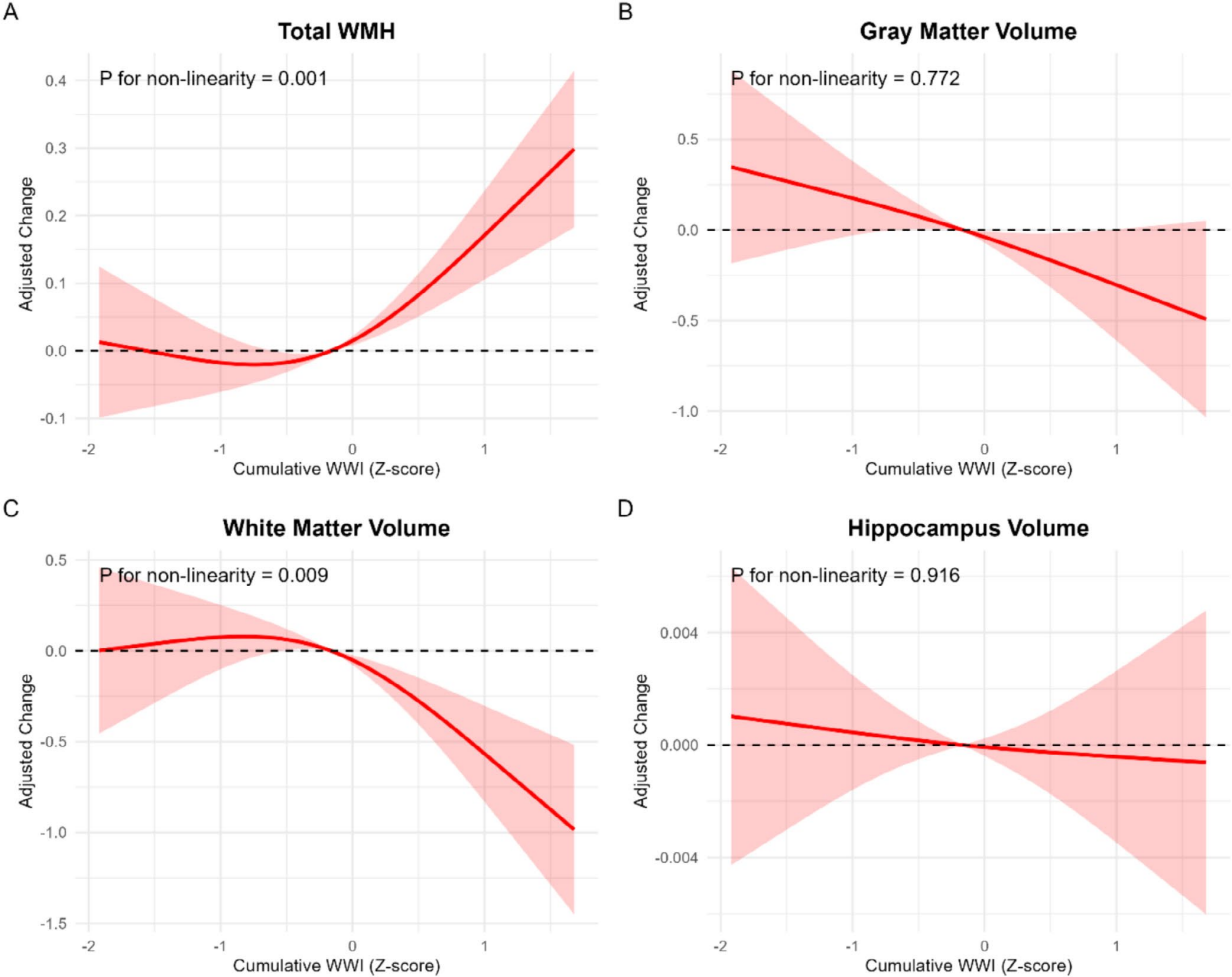
Non‐linear association between Cumulative WWI and Neuroimaging features in Females. Restricted cubic spline (RCS) models with three knots (at the 10th, 50th, and 90th percentiles) were used to explore the continuous relationships between standardized cumulative WWI (*Z*‐score, x‐axis) and adjusted changes in brain structural outcomes (y‐axis). The solid red line represents the estimated adjusted change, and the shaded area indicates the 95% confidence interval. The dashed horizontal line represents the reference level (no change). The models were fully adjusted for age, educational level, smoking status, drinking status, physical activity, systolic blood pressure, diabetes status, LDL‐C, HDL‐C, triglycerides, and history of hypertension. WWI, weight‐adjusted‐waist index.

To analyze the dose–response relationship between WWI and neuroimaging features, we performed RCS analysis. In females, significant non‐linear relationships were observed for multiple markers. Specifically, J‐shaped associations were identified for total WMH (*p* for non‐linearity = 0.001), periventricular WMH (*p* for non‐linearity = 0.003), and deep WMH (*p* for non‐linearity = 0.002), suggesting that the risk of white matter lesion accumulation increases sharply once cumulative WWI exceeds a certain threshold (approximately 9.78 to 9.80 cm/√kg). Furthermore, white matter volume exhibited a non‐linear pattern (*p* for non‐linearity = 0.009), with steeper volume loss at higher WWI levels (Figures 1 and S2). In contrast, linear trends were observed for gray matter volume, hippocampus volume, and other markers.

### 3.5 Regional Brain Macro‐ and Microstructural Associations

**FIGURE 2:**
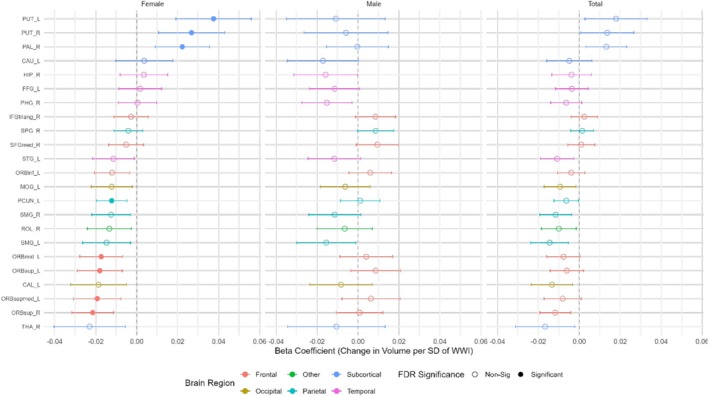
Forest Plot of Brain Substructures Associated with Cumulative WWI. Forest plot showing the beta coefficients (with 95% confidence intervals) from linear regression models evaluating the association between standardized WWI and brain volumes in specific regions of interest (ROIs). Models were adjusted for age, sex (in the total sample), and total intracranial volume (TIV). Results are stratified by the total population, males, and females. Solid circles indicate significant associations after false discovery rate (FDR) correction (*p* < 0.05), while hollow circles indicate non‐significant results. CI, confidence interval; FDR, false discovery rate; WWI, weight‐adjusted‐waist index.

Using the AAL90 atlas, specific regional brain volume changes associated with cumulative WWI were identified. Additionally, regional WMH burden and DTI metrics were examined. After FDR correction, significant associations were observed exclusively in females. Regarding gray matter volumes, cumulative WWI was negatively associated with volumes in the frontal lobe regions, including the right superior orbital gyrus (Beta = −0.021, *p*_FDR_ = 0.003), left superior orbital gyrus (Beta = −0.018, *p*_FDR_ = 0.016), left middle orbital gyrus (Beta = −0.017, *p*_FDR_ = 0.016), and left superior medial orbital gyrus (Beta = −0.019, *p*_FDR_ = 0.016), as well as the left precuneus (Beta = −0.012, *p*_FDR_ = 0.016). Positive associations were observed in the basal ganglia, specifically in the bilateral putamen and right pallidum (Figure 2 and Table S4).

For WMH, cumulative WWI was significantly associated with increased WMH burden across all assessed regions, including the frontal, temporal, occipital, parietal, and limbic lobes, as well as sub‐lobar regions (*p*_FDR_ < 0.05). The strongest associations were observed in the frontal lobes (Left: Beta = 0.107; Right: Beta = 0.097) (Table S5 and Figure S3). In terms of DTI metrics, widespread associations between cumulative WWI and microstructural alterations were found. Specifically, higher cumulative WWI was linked to increased MD in numerous tracts, including projection fibers, association fibers, and limbic system tracts (Table S6 and Figure S4). These findings suggest widespread disruption of white matter integrity. No significant regional associations were found in males.

### 3.6 Exploratory Statistical Mediation Analysis

Exploratory statistical mediation analyzes suggested that FBG, SBP, and hs‐CRP statistically accounted for part of the observed associations between cumulative WWI and white matter markers in females. Specifically, FBG, SBP, and hs‐CRP showed significant potential mediating roles for total WMH. FBG mediated approximately 14.7% of the association (*p* = 0.018), SBP mediated 11.3% (*p* = 0.018), and hs‐CRP mediated 11.3% (*p* = 0.012). SBP also accounted for 17.8% of the association between cumulative WWI and global MD, whereas hs‐CRP accounted for a smaller proportion of this association (Table 4).

**TABLE 4:** Exploratory statistical mediation analysis of metabolic factors in the associations of cumulative WWI with white matter integrity in females.

Outcome	Mediator	Indirect effect (95% CI)	Direct effect (95% CI)	Total effect (95% CI)	Proportion mediated	*p*
Log total WMH	FBG	0.0048 (0.0010, 0.0094)	0.0271 (0.0099, 0.0443)	0.0319 (0.0155, 0.0486)	14.70%	0.018
	SBP	0.0037 (0.0006, 0.0080)	0.0283 (0.0110, 0.0454)	0.0320 (0.0154, 0.0488)	11.30%	0.018
	hs‐CRP	0.0039 (0.0010, 0.0078)	0.0292 (0.0121, 0.0462)	0.0331 (0.0161, 0.0497)	11.30%	0.012
Global mean diffusivity	SBP	0.0224 (0.0088, 0.0392)	0.1010 (0.0400, 0.1612)	0.1234 (0.0644, 0.1824)	17.80%	< 0.001
	FBG	0.0019 (−0.0125, 0.0177)	0.1096 (0.0479, 0.1714)	0.1116 (0.0528, 0.1730)	1.70%	0.790
	hs‐CRP	0.0072 (0.0002, 0.0175)	0.1088 (0.0478, 0.1699)	0.1160 (0.0554, 0.1759)	5.90%	0.040

*Note:* The models were adjusted for age, educational level, smoking status, drinking status, physical activity, systolic blood pressure, diabetes status, LDL‐C, HDL‐C, triglycerides, and history of hypertension.

Abbreviations: CI, confidence interval; FBG, fasting blood glucose; hs‐CRP, high‐sensitivity C‐reactive protein; MD, mean diffusivity; SBP, systolic blood pressure; WMH, white matter hyperintensity; WWI, weight‐adjusted‐waist index.

## 4 Discussion

In this longitudinal cohort study, long‐term exposure to WWI was identified as a potential marker for adverse brain structural outcomes in mid‐to‐late life. The findings reveal a distinct sex‐specific vulnerability, where elevated cumulative WWI in females was associated with extensive WMH accumulation, widespread microstructural disintegration, and focal gray matter atrophy predominantly in the frontal lobes. Furthermore, exploratory mediation analyzes provided hypothesis‐generating evidence that glycemic, inflammatory, and hemodynamic factors statistically accounted for part of the observed associations. These results suggest that cumulative WWI may serve as a potential marker for cerebrovascular and neurodegenerative pathology.

The age‐stratified analysis revealed that the adverse association of cumulative WWI was more pronounced in the midlife group (< 60 years) compared to older adults. This discrepancy aligns perfectly with the well‐established “midlife obesity” hypothesis in neurodegeneration. In midlife, central adiposity and metabolic dysregulation may act as primary contributors to systemic inflammation and microvascular damage. However, in older adults (≥ 60 years), the overarching influence of normal biological aging and accumulated neurodegenerative pathologies likely overpower the specific contribution of adiposity. Additionally, survival bias or the “obesity paradox” in late life might dilute the observed associations in the older stratum.

A key finding of our study is the vulnerability of females to high cumulative WWI, particularly concerning white matter microstructural integrity. This sex discrepancy likely stems from a complex interplay of hormonal, adipose distribution, and inflammatory factors driven by the menopausal transition [22]. First, estrogen plays a well‐established neuroprotective role and regulates adipose tissue distribution [23, 24]. The postmenopausal withdrawal of sex hormones predisposes women to a distinct shift from subcutaneous to visceral fat accumulation [25], which is highly metabolically active and lipotoxic [25]. Consequently, an elevated WWI in mid‐to‐late life females may reflect a more severe and sudden accumulation of this detrimental visceral fat compared to the more gradual changes observed in males. Second, this visceral adiposity strongly exacerbates systemic inflammation [26]. Without the immunomodulatory and dampening effects of estrogen on microglial activation, postmenopausal females may exhibit heightened neuroinflammatory responses and greater microvascular vulnerability to adiposity‐induced systemic inflammation [27]. This increased susceptibility may allow systemic inflammatory cytokines to compromise the blood–brain barrier more easily, leading to more extensive white matter demyelination and microstructural damage. However, these underlying mechanisms require further experimental validation.

The findings of the current study extend the growing body of literature on WWI and health outcomes. While previous studies have linked WWI to all‐cause mortality [28] and cardiovascular risk [29, 30], and incident stroke in older adults with hypertension [31], this study is among the first to demonstrate its association with brain structure. Previous studies using BMI have yielded conflicting results regarding brain health, likely due to BMI's inability to distinguish between fat and muscle mass [4]. WWI has been reported to correlate positively with fat mass and negatively with muscle mass [11]; however, in the absence of direct body composition measurements, it should be interpreted as an anthropometric marker of adverse body composition rather than a validated measure of sarcopenic obesity. These results align with recent findings from the UK Biobank [32] and other cohorts [16], which suggest that central adiposity is a more potent predictor for brain aging than general obesity. However, unlike cross‐sectional studies that rely on a single measurement, the present study utilized a 12‐year cumulative exposure model, providing stronger evidence for a temporal association. The sensitivity analyzes, including exclusion of participants with hypertension, diabetes, or cardiovascular disease, generally supported the robustness of the main findings. Because BMI and WWI both incorporate body weight, additional BMI adjustment may introduce over‐adjustment and should be interpreted cautiously. Therefore, the BMI‐adjusted model was considered exploratory and was not used as the primary inferential model. In this analysis, the associations with white matter injury remained directionally consistent but were attenuated after FDR correction.

Furthermore, our findings extend earlier neuroimaging studies that used conventional measures of central adiposity, such as waist circumference, waist‐to‐hip ratio, or visceral adipose tissue imaging. Previous studies have linked abdominal adiposity to brain atrophy, altered brain aging patterns, and white matter injury [32, 33, 34, 35, 36]. However, most prior studies relied on single‐time‐point adiposity assessments. By incorporating repeated anthropometric measurements over a 12‐year period, our study captures the cumulative burden of adverse adiposity‐related exposure before MRI acquisition. This longitudinal exposure framework may help explain the observed associations with white matter injury and frontal gray matter alterations.

Mechanistically, adiposity‐related inflammation may provide a more directly supported explanation for these findings. Visceral and central adiposity are closely linked to systemic inflammation, endothelial dysfunction, blood–brain barrier disruption, and cerebral small vessel disease, all of which may contribute to WMH accumulation and white matter microstructural injury [19, 26, 37, 38, 39, 40, 41, 42]. In our exploratory mediation analyzes, hs‐CRP statistically accounted for part of the association between cumulative WWI and WMH burden, supporting the potential involvement of systemic inflammation. Although previous studies have reported that WWI correlates positively with fat mass and negatively with muscle mass [10, 11], direct measures of muscle mass, visceral fat, or body composition imaging were unavailable in the present study. Therefore, WWI should be interpreted as a practical anthropometric marker related to central adiposity and adverse body composition, rather than as a validated measure of sarcopenic obesity.

A novel finding of this study is the J‐shaped dose–response relationship between cumulative WWI and WMH burden in females. The risk of high WMH burden increases sharply once WWI exceeds a certain threshold, suggesting a tipping point where metabolic and inflammatory resilience is overwhelmed [36]. This non‐linear pattern was also observed for white matter volume loss. In contrast, gray matter atrophy followed a linear trajectory, implying that neuronal loss may track more directly with the continuous severity of central obesity. These findings highlight the importance of early intervention to keep WWI below the critical threshold to prevent extensive white matter injury.

Our regional analysis revealed a striking vulnerability of the frontal lobes in relation to cumulative WWI. We observed both significant gray matter atrophy in the orbital frontal cortex and the highest burden of WMH in the frontal region. The frontal lobes are critical for executive function and are known to be highly sensitive to metabolic and vascular dysregulation [43]. The concordance of gray matter loss and white matter lesions in this region suggests a localized susceptibility, possibly due to the unique vascular architecture or metabolic demands of the prefrontal cortex. Additionally, we observed increased volume in the basal ganglia (putamen and pallidum). While typically atrophy is expected, increased volume in these structures has been reported in early stages of neuroinflammation or metabolic disruption, potentially reflecting glial activation or osmotic swelling. The widespread microstructural DTI across projection and association fibers further corroborates a systemic failure of white matter integrity.

Exploratory statistical mediation analyzes suggested that different metabolic and vascular factors may statistically account for different aspects of brain injury. FBG, SBP, and hs‐CRP statistically accounted for part of the association between cumulative WWI and WMH burden, supporting the hypothesis that glycemic dysregulation, hemodynamic stress, and systemic inflammation jointly contribute to the development of macroscopic white matter lesions [38]. Hyperglycemia and insulin resistance are known to damage the endothelial lining of cerebral small vessels, leading to blood–brain barrier (BBB) leakage [39, 40], while systemic inflammation can further compromise the BBB and activate microglia [38]. In contrast, SBP emerged as the predominant mediator for global MD, accounting for 17.8% of the total association, with hs‐CRP playing a minor secondary role (5.9%). We interpret this through the lens of a “multi‐hit” model: metabolic and systemic inflammatory pathways combined with blood pressure may primarily contribute to endothelial dysfunction leading to macroscopic small vessel disease [41], whereas chronic hemodynamic overload (SBP) might be more directly associated with subtle, diffuse microstructural disintegration via mechanical stress [42].

Given its low cost and accessibility—requiring only a tape measure and a scale—tracking WWI trajectories may serve as a highly practical screening tool in clinical and primary care settings. While current guidelines for obesity management primarily focus on BMI [44], our findings suggest that WC and its adjustment for weight (WWI) provide crucial added prognostic value and should be integrated into routine geriatric and cardiovascular risk assessments. Identifying individuals with high WWI in midlife (particularly females approaching or exceeding the 9.8 cm/√kg threshold) could trigger early, targeted interventions. Even if a patient's BMI falls within the normal range, clinicians should be vigilant about metabolic screening and consider lifestyle interventions focusing on visceral fat reduction and muscle mass maintenance. For instance, high‐intensity resistance training has been shown to be effective for central adiposity and may offer neuroprotective benefits [45, 46]. Such interventions might yield the most significant benefits when initiated in midlife, which represents a critical window of opportunity for dementia prevention [47]. Ultimately, establishing clear, actionable clinical WWI thresholds and confirming the neuroprotective efficacy of these interventions must await future prospective interventional trials.

Strengths of this study include the prospective longitudinal design with a 12‐year exposure window, which allowed for the assessment of cumulative WWI. This approach captures the chronic burden of central obesity more effectively than cross‐sectional measurements. The use of WWI provides a practical longitudinal anthropometric marker related to central adiposity and adverse body composition. Furthermore, the comprehensive multi‐modal MRI assessment provided a detailed mapping of brain structural integrity, from macrostructural atrophy to microstructural white matter injury. Several limitations should be noted. First, due to the observational design, while we have made every effort to include a comprehensive array of potential influencing factors in our models, it is inherently impossible to capture every relevant variable; thus, residual confounding from unmeasured factors cannot be entirely ruled out. Second, the absence of baseline neuroimaging precludes the direct measurement of longitudinal atrophy rates, but our study leverages up to 12 years of prospective WWI tracking prior to the MRI assessment. This unique design establishes a clear temporal sequence that substantially mitigates reverse causation, thereby yielding more robust structural associations than conventional cross‐sectional studies. Third, mediators and neuroimaging outcomes were assessed concurrently, precluding definitive causal inference; therefore, the mediation findings should be interpreted as hypothesis‐generating. Fourth, without direct body composition imaging, we cannot definitively disentangle the precise contributions of sarcopenic obesity versus central adiposity alone. Finally, the Kailuan cohort predominantly consists of a northern Chinese population, which may limit generalizability. In addition, excluded participants were relatively younger, suggesting possible underrepresentation of working‐age individuals. This differential attrition may have influenced the estimation of midlife‐specific associations and the apparent WWI threshold observed in the spline analyzes. Therefore, the age‐stratified and threshold findings should be interpreted cautiously and require validation in larger cohorts with more complete follow‐up.

## 5 Conclusion

In conclusion, our findings suggest that cumulative WWI may serve as a significant indicator of brain structural integrity, with elevated levels potentially linked to white matter injury, particularly in females. Exploratory mediation analyzes suggested that glycemic, hemodynamic, and inflammatory factors may statistically account for part of these associations. These findings support the potential value of monitoring long‐term central adiposity‐related anthropometric profiles in midlife as part of broader strategies to preserve brain health.

## Author Contributions

**Qi Sun:** conceptualization, methodology, writing – original draft, writing – review and editing. **Li Yu:** data curation, methodology, writing – review and editing. **Ling Yang:** formal analysis, investigation. **Ying Hui:** investigation, resources. **Mingze Xu:** methodology, software. **Yanbo Liang:** formal analysis, validation. **Qunya Qi:** visualization, data curation. **Zhenchang Wang:** supervision, resources. **Shouling Wu:** resources, investigation. **Yuntao Wu:** conceptualization, supervision, writing – review and editing. **Han Lv:** project administration, conceptualization, writing – review and editing.

## Funding

This work was supported by the National Natural Science Foundation of China (Grant 52500256 and Grant 62522119), and Beijing High‐Level Innovation and Entrepreneurship Talent Support Program, young top talent projects (Grant G202522110).

## Ethics Statement

The protocol for this research received formal approval from the Medical Ethics Committee of Kailuan General Hospital (No: 2021002).

## Consent

Prior to their inclusion, all participants provided written informed consent. No financial stipend was offered for participation.

## Conflicts of Interest

The authors declare no conflicts of interest.

## Supporting information


**Table S1:** Baseline characteristics comparison between the included analytic sample and excluded participants.
**Table S2:** Exploratory sensitivity analyzes of the association between cumulative WWI and neuroimaging features.
**Table S3:** Age‐stratified association between cumulative WWI and neuroimaging markers.
**Table S4:** Multivariable associations between cumulative WWI and volumes of 90 brain substructures.
**Table S5:** Associations of cumulative WWI with regional white matter hyperintensity volumes.
**Table S6:** Associations of cumulative WWI with regional diffusion tensor imaging metrics (FA and MD).
**Figure S1:** Flowchart of participant selection.
**Figure S2:** Dose–response relationships between the weight‐adjusted‐waist index (WWI) and neuroimaging markers of brain health.
**Figure S3:** Association of the weight‐adjusted‐waist index with regional white matter hyperintensities.
**Figure S4:** Association of the weight‐adjusted‐waist index with diffusion tensor imaging metrics.

## Data Availability

The data that support the findings of this study are available on request from the corresponding author. The data are not publicly available due to privacy or ethical restrictions.
