# Supplementary material for: Association of Weight‐Adjusted‐Waist Index With Brain Health: A 16‐Year Population‐Based Longitudinal Cohort Study
Source: CNS Neurosci Ther. 2026 Jun 23;32(6):e71001. doi: 10.1002/cns.71001 (PMC13289582; doi:10.1002/cns.71001)
Supplement: Supplementary file 1 — Table S1: Baseline characteristics comparison between the included analytic sample and excluded participants. Table S2: Exploratory sensitivity analyzes of the association between cumulative WWI and neuroimaging features. Table S3: Age‐stratified association between cumulative WWI and neuroimaging markers. Table S4: Multivariable associations between cumulative WWI and volumes of 90 brain substructures. Table S5: Associations of cumulative WWI with regional white matter hyperintensity volumes. Table S6: Associations of cumulative WWI with regional diffusion tensor imaging metrics (FA and MD). Figure S1: Flowchart of participant selection. Figure S2: Dose–response relationships between the weight‐adjusted‐waist index (WWI) and neuroimaging markers of brain health. Figure S3: Association of the weight‐adjusted‐waist index with regional white matter hyperintensities. Figure S4: Association of the weight‐adjusted‐waist index with diffusion tensor imaging metrics. [file CNS-32-e71001-s001.docx]

Supplementary Table S1. Baseline characteristics comparison between the included analytic sample and excluded participants.

| Characteristics | Included (N = 935) | Excluded (N = 246) | *P*-value |
| --- | --- | --- | --- |
| Age at MRI (years), mean (SD) | 56.02 (11.01) | 51.61 (12.98) | <0.001 |
| Sex, n (%) |  |  | 0.335 |
| Female | 312 (46.0%) | 33 (39.8%) |  |
| Male | 366 (54.0%) | 50 (60.2%) |  |
| Education Level, n (%) |  |  | 0.921 |
| Low | 305 (45.2%) | 39 (46.4%) |  |
| High | 370 (54.8%) | 45 (53.6%) |  |
| Smoking History, n (%) |  |  | 0.060 |
| Never | 513 (73.1%) | 56 (62.9%) |  |
| Ever | 189 (26.9%) | 33 (37.1%) |  |
| Drinking History, n (%) |  |  | 0.791 |
| Never | 417 (59.4%) | 51 (57.3%) |  |
| Ever | 285 (40.6%) | 38 (42.7%) |  |
| Physical Activity, n (%) |  |  | 0.825 |
| None | 41 (6.1%) | 6 (7.1%) |  |
| Occasional | 572 (84.7%) | 69 (82.1%) |  |
| Frequent | 62 (9.2%) | 9 (10.7%) |  |
| Body Mass Index (kg/m²), mean (SD) | 24.59 (3.45) | 24.77 (3.11) | 0.639 |
| Systolic Blood Pressure (mmHg), mean (SD) | 118.10 (16.45) | 120.29 (16.35) | 0.234 |
| Fasting Blood Glucose (mmol/L), mean (SD) | 5.17 (1.38) | 5.32 (1.47) | 0.314 |
| LDL-C (mmol/L), mean (SD) | 2.23 (0.76) | 2.26 (0.66) | 0.702 |
| HDL-C (mmol/L), mean (SD) | 1.50 (0.35) | 1.41 (0.34) | 0.021 |

*Note:* SD, standard deviation; MRI, magnetic resonance imaging; LDL-C, low-density lipoprotein cholesterol; HDL-C, high-density lipoprotein cholesterol.

Supplementary Table S2. Exploratory sensitivity analyses of the association between cumulative WWI and neuroimaging features.

| Outcome | Exposure | Model 1 | | Model 2 | | Model 3 | |
| --- | --- | --- | --- | --- | --- | --- | --- |
|  |  | Beta (95% CI) | *P* (P_FDR_) | Beta (95% CI) | *P* (P_FDR_) | Beta (95% CI) | *P* (P_FDR_) |
| Total Brain Volume | Medium vs Low | 0.441 (-0.038, 0.920) | 0.071 (0.160) | -0.147 (-0.610, 0.315) | 0.532 (0.821) | 0.016 (-0.432, 0.464) | 0.945 (0.981) |
| Total Brain Volume | High vs Low | -0.303 (-0.804, 0.197) | 0.235 (0.392) | -0.878 (-1.358, -0.399) | <0.001 (0.001) | -0.777 (-1.239, -0.315) | 0.001 (0.004) |
| Gray Matter Volume | Medium vs Low | 0.300 (-0.028, 0.629) | 0.074 (0.160) | -0.092 (-0.411, 0.226) | 0.570 (0.821) | 0.009 (-0.297, 0.315) | 0.955 (0.981) |
| Gray Matter Volume | High vs Low | 0.095 (-0.249, 0.438) | 0.589 (0.625) | -0.297 (-0.627, 0.033) | 0.078 (0.174) | -0.227 (-0.543, 0.089) | 0.159 (0.353) |
| White Matter Volume | Medium vs Low | 0.141 (-0.185, 0.468) | 0.397 (0.496) | -0.055 (-0.351, 0.241) | 0.716 (0.821) | 0.007 (-0.278, 0.291) | 0.962 (0.981) |
| White Matter Volume | High vs Low | -0.398 (-0.739, -0.057) | 0.022 (0.112) | -0.582 (-0.889, -0.275) | <0.001 (0.001) | -0.550 (-0.843, -0.256) | <0.001 (0.001) |
| CSF Volume | Medium vs Low | -0.421 (-0.892, 0.050) | 0.080 (0.160) | 0.154 (-0.303, 0.610) | 0.510 (0.821) | -0.005 (-0.446, 0.436) | 0.981 (0.981) |
| CSF Volume | High vs Low | 0.264 (-0.228, 0.756) | 0.293 (0.451) | 0.847 (0.373, 1.321) | <0.001 (0.001) | 0.743 (0.289, 1.198) | 0.001 (0.004) |
| Hippocampal Volume | Medium vs Low | 0.001 (-0.002, 0.005) | 0.469 (0.552) | 0.001 (-0.003, 0.004) | 0.706 (0.821) | 0.001 (-0.003, 0.004) | 0.703 (0.939) |
| Hippocampal Volume | High vs Low | -0.002 (-0.006, 0.002) | 0.388 (0.496) | -0.004 (-0.008, -0.001) | 0.014 (0.035) | -0.004 (-0.007, -0.000) | 0.036 (0.089) |
| Total WMH Volume | Medium vs Low | -0.122 (-0.254, 0.010) | 0.071 (0.160) | -0.017 (-0.115, 0.080) | 0.728 (0.821) | -0.068 (-0.187, 0.050) | 0.259 (0.471) |
| Total WMH Volume | High vs Low | 0.170 (0.032, 0.309) | 0.016 (0.106) | 0.234 (0.133, 0.334) | <0.001 (<0.001) | 0.232 (0.110, 0.353) | <0.001 (0.001) |
| Periventricular WMH | Medium vs Low | -0.039 (-0.096, 0.017) | 0.173 (0.315) | -0.002 (-0.044, 0.040) | 0.913 (0.913) | -0.018 (-0.067, 0.032) | 0.486 (0.747) |
| Periventricular WMH | High vs Low | 0.086 (0.026, 0.145) | 0.005 (0.094) | 0.113 (0.070, 0.156) | <0.001 (<0.001) | 0.103 (0.053, 0.154) | <0.001 (0.001) |
| Deep WMH | Medium vs Low | -0.082 (-0.168, 0.003) | 0.058 (0.160) | -0.015 (-0.079, 0.049) | 0.645 (0.821) | -0.051 (-0.128, 0.026) | 0.196 (0.392) |
| Deep WMH | High vs Low | 0.084 (-0.005, 0.173) | 0.064 (0.160) | 0.120 (0.054, 0.186) | <0.001 (0.001) | 0.128 (0.049, 0.207) | 0.002 (0.004) |
| Global FA | Medium vs Low | 0.008 (-0.170, 0.187) | 0.928 (0.928) | 0.026 (-0.126, 0.178) | 0.738 (0.821) | 0.028 (-0.116, 0.172) | 0.705 (0.939) |
| Global FA | High vs Low | -0.081 (-0.268, 0.105) | 0.394 (0.496) | -0.090 (-0.248, 0.068) | 0.264 (0.528) | -0.071 (-0.220, 0.078) | 0.348 (0.579) |
| Global MD | Medium vs Low | -0.040 (-0.187, 0.107) | 0.593 (0.625) | 0.010 (-0.114, 0.133) | 0.876 (0.913) | 0.008 (-0.116, 0.132) | 0.900 (0.981) |
| Global MD | High vs Low | 0.198 (0.044, 0.351) | 0.012 (0.106) | 0.271 (0.143, 0.399) | <0.001 (<0.001) | 0.251 (0.122, 0.379) | <0.001 (0.001) |

WWI = Weight-adjusted-waist Index; CI = Confidence Interval; WMH = White Matter Hyperintensity; FA = Fractional Anisotropy; MD = Mean Diffusivity; CSF = Cerebrospinal Fluid. Model 1 additionally adjusted the fully adjusted model for BMI as an exploratory sensitivity analysis; because BMI and WWI both incorporate body weight, these results should be interpreted cautiously. Model 2: Replicated the main analysis after excluding participants with a history of hypertension (HTN) or diabetes mellitus (DM). Model 3: Replicated the main analysis after excluding participants with a history of cardiovascular disease (CVD). *P* represents the raw *P*-value, and P_FDR_ represents the False Discovery Rate-corrected *P*-value adjusting for multiple testing across all neuroimaging outcomes.

Supplementary Table S3. Age-Stratified Association between Cumulative WWI and Neuroimaging Markers

| Outcome | WWI Tertile | Age < 60 years | | Age ≥ 60 years | | *P* for Interaction |
| --- | --- | --- | --- | --- | --- | --- |
|  |  | β (95% CI) | *P* (P_FDR_) | β (95% CI) | *P* (P_FDR_) |  |
| Brain Parenchyma Volume | Low | Ref. |  | Ref. |  | 0.885 |
|  | Medium | -0.023 (-0.538, 0.491) | 0.929 (0.952) | 0.195 (-0.676, 1.066) | 0.661 (0.806) |  |
|  | High | -0.914 (-1.470, -0.358) | 0.001 (0.005) | -0.429 (-1.269, 0.412) | 0.318 (0.806) |  |
| Gray Matter Volume | Low | Ref. |  | Ref. |  | 0.853 |
|  | Medium | -0.044 (-0.416, 0.329) | 0.818 (0.952) | 0.085 (-0.481, 0.650) | 0.769 (0.810) |  |
|  | High | -0.284 (-0.686, 0.119) | 0.168 (0.420) | -0.137 (-0.683, 0.409) | 0.623 (0.806) |  |
| White Matter Volume | Low | Ref. |  | Ref. |  | 0.838 |
|  | Medium | 0.020 (-0.299, 0.340) | 0.900 (0.952) | 0.110 (-0.453, 0.674) | 0.702 (0.806) |  |
|  | High | -0.630 (-0.976, -0.285) | <0.001 (0.002) | -0.292 (-0.836, 0.252) | 0.294 (0.806) |  |
| CSF Volume | Low | Ref. |  | Ref. |  | 0.826 |
|  | Medium | 0.024 (-0.486, 0.535) | 0.926 (0.952) | -0.201 (-1.050, 0.649) | 0.644 (0.806) |  |
|  | High | 0.892 (0.340, 1.444) | 0.002 (0.005) | 0.367 (-0.453, 1.187) | 0.381 (0.806) |  |
| Hippocampus Volume | Low | Ref. |  | Ref. |  | 0.160 |
|  | Medium | -0.000 (-0.004, 0.003) | 0.897 (0.952) | 0.002 (-0.004, 0.008) | 0.548 (0.806) |  |
|  | High | -0.002 (-0.006, 0.002) | 0.297 (0.594) | -0.004 (-0.010, 0.002) | 0.173 (0.759) |  |
| Total WMH Volume | Low | Ref. |  | Ref. |  | 0.074 |
|  | Medium | 0.005 (-0.068, 0.079) | 0.889 (0.952) | -0.089 (-0.407, 0.229) | 0.584 (0.806) |  |
|  | High | 0.184 (0.106, 0.263) | <0.001 (<0.001) | 0.258 (-0.049, 0.565) | 0.101 (0.674) |  |
| Periventricular WMH | Low | Ref. |  | Ref. |  | 0.010 |
|  | Medium | 0.016 (-0.013, 0.045) | 0.284 (0.594) | -0.032 (-0.163, 0.099) | 0.632 (0.806) |  |
|  | High | 0.071 (0.040, 0.103) | <0.001 (<0.001) | 0.124 (-0.003, 0.250) | 0.056 (0.674) |  |
| Deep WMH | Low | Ref. |  | Ref. |  | 0.295 |
|  | Medium | -0.011 (-0.062, 0.040) | 0.676 (0.952) | -0.057 (-0.263, 0.150) | 0.592 (0.806) |  |
|  | High | 0.113 (0.058, 0.167) | <0.001 (<0.001) | 0.134 (-0.066, 0.334) | 0.190 (0.759) |  |
| Global FA | Low | Ref. |  | Ref. |  | 0.492 |
|  | Medium | 0.004 (-0.120, 0.128) | 0.952 (0.952) | 0.065 (-0.296, 0.426) | 0.725 (0.806) |  |
|  | High | -0.043 (-0.178, 0.091) | 0.529 (0.881) | -0.077 (-0.426, 0.273) | 0.668 (0.806) |  |
| Global MD | Low | Ref. |  | Ref. |  | 0.177 |
|  | Medium | 0.050 (-0.062, 0.162) | 0.380 (0.691) | -0.035 (-0.329, 0.260) | 0.818 (0.818) |  |
|  | High | 0.193 (0.071, 0.314) | 0.002 (0.006) | 0.254 (-0.031, 0.539) | 0.082 (0.674) |  |

Note: WWI, weight-adjusted-waist index; CI, confidence interval; Ref, reference; WMH, white matter hyperintensity; FA, fractional anisotropy; MD, mean diffusivity; CSF, cerebrospinal fluid. The results present the fully adjusted model, which controlled for age (as a continuous variable within strata), sex, educational level, smoking status, drinking status, physical activity, systolic blood pressure, diabetes status, LDL-C, HDL-C, triglycerides, and history of hypertension. P represents the raw P-value, and P_FDR_ represents the FDR-corrected P-value adjusted across the 10 predefined neuroimaging outcomes separately within each age-stratified model.

Supplementary Table S4. Multivariable associations between cumulative WWI and volumes of 90 brain substructures

| Group | Outcome | Beta (95% CI) | *P* | P_FDR_ |
| --- | --- | --- | --- | --- |
| Total | PreCG_L | 0.000 (-0.006, 0.007) | 0.886 | 0.941 |
| Total | PreCG_R | 0.002 (-0.005, 0.008) | 0.623 | 0.795 |
| Total | SFGdor_L | 0.000 (-0.005, 0.006) | 0.87 | 0.941 |
| Total | SFGdor_R | 0.001 (-0.004, 0.006) | 0.788 | 0.898 |
| Total | ORBsup_L | -0.006 (-0.014, 0.002) | 0.144 | 0.378 |
| Total | ORBsup_R | -0.012 (-0.019, -0.004) | 0.002 | 0.111 |
| Total | MFG_L | -0.003 (-0.009, 0.004) | 0.409 | 0.671 |
| Total | MFG_R | -0.003 (-0.009, 0.003) | 0.352 | 0.621 |
| Total | ORBmid_L | -0.008 (-0.016, 0.001) | 0.066 | 0.246 |
| Total | ORBmid_R | -0.008 (-0.016, -0.000) | 0.045 | 0.225 |
| Total | IFGoperc_L | 0.002 (-0.005, 0.008) | 0.65 | 0.801 |
| Total | IFGoperc_R | -0.002 (-0.010, 0.005) | 0.538 | 0.766 |
| Total | IFGtriang_L | 0.001 (-0.007, 0.008) | 0.888 | 0.941 |
| Total | IFGtriang_R | 0.002 (-0.004, 0.009) | 0.455 | 0.706 |
| Total | ORBinf_L | -0.004 (-0.011, 0.003) | 0.247 | 0.505 |
| Total | ORBinf_R | -0.004 (-0.010, 0.002) | 0.205 | 0.473 |
| Total | ROL_L | -0.008 (-0.016, 0.001) | 0.068 | 0.246 |
| Total | ROL_R | -0.010 (-0.019, -0.001) | 0.021 | 0.215 |
| Total | SMA_L | -0.000 (-0.007, 0.007) | 0.999 | 0.999 |
| Total | SMA_R | 0.000 (-0.006, 0.007) | 0.944 | 0.966 |
| Total | OLF_L | -0.005 (-0.015, 0.005) | 0.328 | 0.594 |
| Total | OLF_R | -0.008 (-0.018, 0.001) | 0.096 | 0.288 |
| Total | SFGmed_L | 0.000 (-0.005, 0.005) | 0.924 | 0.956 |
| Total | SFGmed_R | 0.001 (-0.006, 0.008) | 0.774 | 0.895 |
| Total | ORBsupmed_L | -0.008 (-0.017, 0.001) | 0.08 | 0.257 |
| Total | ORBsupmed_R | -0.010 (-0.020, -0.000) | 0.042 | 0.225 |
| Total | REC_L | -0.003 (-0.011, 0.005) | 0.41 | 0.671 |
| Total | REC_R | -0.005 (-0.014, 0.005) | 0.315 | 0.591 |
| Total | INS_L | -0.003 (-0.012, 0.006) | 0.522 | 0.757 |
| Total | INS_R | -0.009 (-0.018, -0.000) | 0.039 | 0.225 |
| Total | ACG_L | -0.007 (-0.016, 0.003) | 0.183 | 0.434 |
| Total | ACG_R | -0.009 (-0.020, 0.001) | 0.074 | 0.246 |
| Total | DCG_L | -0.005 (-0.013, 0.003) | 0.222 | 0.475 |
| Total | DCG_R | -0.007 (-0.014, 0.000) | 0.068 | 0.246 |
| Total | PCG_L | -0.012 (-0.022, -0.001) | 0.035 | 0.225 |
| Total | PCG_R | -0.005 (-0.012, 0.002) | 0.18 | 0.434 |
| Total | HIP_L | -0.006 (-0.017, 0.005) | 0.279 | 0.558 |
| Total | HIP_R | -0.004 (-0.013, 0.006) | 0.44 | 0.695 |
| Total | PHG_L | 0.002 (-0.004, 0.008) | 0.579 | 0.789 |
| Total | PHG_R | -0.006 (-0.014, 0.001) | 0.1 | 0.291 |
| Total | AMYG_L | -0.001 (-0.011, 0.010) | 0.92 | 0.956 |
| Total | AMYG_R | 0.001 (-0.009, 0.011) | 0.838 | 0.941 |
| Total | CAL_L | -0.013 (-0.023, -0.003) | 0.01 | 0.151 |
| Total | CAL_R | -0.009 (-0.019, 0.001) | 0.072 | 0.246 |
| Total | CUN_L | -0.000 (-0.008, 0.008) | 0.989 | 0.999 |
| Total | CUN_R | 0.002 (-0.007, 0.010) | 0.682 | 0.829 |
| Total | LING_L | -0.002 (-0.009, 0.005) | 0.545 | 0.766 |
| Total | LING_R | -0.004 (-0.011, 0.002) | 0.169 | 0.422 |
| Total | SOG_L | -0.003 (-0.010, 0.003) | 0.286 | 0.56 |
| Total | SOG_R | -0.002 (-0.009, 0.005) | 0.562 | 0.779 |
| Total | MOG_L | -0.009 (-0.017, -0.002) | 0.018 | 0.215 |
| Total | MOG_R | -0.006 (-0.013, 0.002) | 0.147 | 0.378 |
| Total | IOG_L | -0.009 (-0.018, 0.000) | 0.06 | 0.246 |
| Total | IOG_R | 0.002 (-0.007, 0.012) | 0.627 | 0.795 |
| Total | FFG_L | -0.004 (-0.012, 0.004) | 0.374 | 0.647 |
| Total | FFG_R | -0.006 (-0.013, 0.001) | 0.096 | 0.288 |
| Total | PoCG_L | -0.004 (-0.010, 0.001) | 0.126 | 0.344 |
| Total | PoCG_R | -0.005 (-0.010, 0.001) | 0.105 | 0.296 |
| Total | SPG_L | -0.002 (-0.009, 0.004) | 0.484 | 0.738 |
| Total | SPG_R | 0.001 (-0.004, 0.007) | 0.619 | 0.795 |
| Total | IPL_L | -0.004 (-0.011, 0.004) | 0.314 | 0.591 |
| Total | IPL_R | -0.001 (-0.010, 0.007) | 0.776 | 0.895 |
| Total | SMG_L | -0.014 (-0.024, -0.005) | 0.002 | 0.111 |
| Total | SMG_R | -0.012 (-0.019, -0.004) | 0.004 | 0.115 |
| Total | ANG_L | -0.006 (-0.016, 0.004) | 0.217 | 0.475 |
| Total | ANG_R | -0.003 (-0.011, 0.005) | 0.426 | 0.685 |
| Total | PCUN_L | -0.006 (-0.012, -0.000) | 0.039 | 0.225 |
| Total | PCUN_R | -0.003 (-0.010, 0.003) | 0.33 | 0.594 |
| Total | PCL_L | 0.002 (-0.006, 0.010) | 0.615 | 0.795 |
| Total | PCL_R | 0.002 (-0.007, 0.011) | 0.691 | 0.829 |
| Total | CAU_L | -0.005 (-0.016, 0.006) | 0.388 | 0.659 |
| Total | CAU_R | 0.002 (-0.010, 0.015) | 0.729 | 0.864 |
| Total | PUT_L | 0.018 (0.003, 0.033) | 0.02 | 0.215 |
| Total | PUT_R | 0.014 (0.001, 0.027) | 0.04 | 0.225 |
| Total | PAL_L | 0.008 (-0.001, 0.016) | 0.071 | 0.246 |
| Total | PAL_R | 0.013 (0.003, 0.023) | 0.01 | 0.151 |
| Total | THA_L | -0.016 (-0.030, -0.001) | 0.035 | 0.225 |
| Total | THA_R | -0.017 (-0.031, -0.002) | 0.025 | 0.223 |
| Total | HES_L | -0.012 (-0.025, 0.001) | 0.063 | 0.246 |
| Total | HES_R | -0.012 (-0.025, 0.001) | 0.072 | 0.246 |
| Total | STG_L | -0.011 (-0.019, -0.003) | 0.009 | 0.151 |
| Total | STG_R | -0.002 (-0.008, 0.004) | 0.521 | 0.757 |
| Total | TPOsup_L | -0.000 (-0.006, 0.005) | 0.878 | 0.941 |
| Total | TPOsup_R | -0.004 (-0.011, 0.002) | 0.212 | 0.475 |
| Total | MTG_L | -0.004 (-0.011, 0.003) | 0.237 | 0.495 |
| Total | MTG_R | 0.002 (-0.005, 0.008) | 0.625 | 0.795 |
| Total | TPOmid_L | -0.001 (-0.010, 0.009) | 0.885 | 0.941 |
| Total | TPOmid_R | -0.008 (-0.015, -0.001) | 0.033 | 0.225 |
| Total | ITG_L | -0.002 (-0.009, 0.005) | 0.648 | 0.801 |
| Total | ITG_R | 0.002 (-0.004, 0.009) | 0.51 | 0.757 |
| Male | PreCG_L | 0.006 (-0.004, 0.016) | 0.237 | 0.916 |
| Male | PreCG_R | 0.004 (-0.005, 0.014) | 0.351 | 0.916 |
| Male | SFGdor_L | 0.004 (-0.004, 0.012) | 0.332 | 0.916 |
| Male | SFGdor_R | 0.007 (-0.002, 0.015) | 0.11 | 0.71 |
| Male | ORBsup_L | 0.009 (-0.004, 0.021) | 0.164 | 0.867 |
| Male | ORBsup_R | 0.001 (-0.011, 0.012) | 0.897 | 0.994 |
| Male | MFG_L | 0.001 (-0.009, 0.011) | 0.875 | 0.994 |
| Male | MFG_R | 0.003 (-0.007, 0.012) | 0.57 | 0.932 |
| Male | ORBmid_L | 0.004 (-0.009, 0.017) | 0.542 | 0.929 |
| Male | ORBmid_R | -0.002 (-0.015, 0.010) | 0.71 | 0.971 |
| Male | IFGoperc_L | 0.007 (-0.004, 0.018) | 0.183 | 0.914 |
| Male | IFGoperc_R | 0.001 (-0.012, 0.014) | 0.887 | 0.994 |
| Male | IFGtriang_L | 0.004 (-0.007, 0.015) | 0.452 | 0.923 |
| Male | IFGtriang_R | 0.009 (-0.001, 0.018) | 0.086 | 0.698 |
| Male | ORBinf_L | 0.006 (-0.004, 0.016) | 0.261 | 0.916 |
| Male | ORBinf_R | 0.002 (-0.008, 0.012) | 0.699 | 0.971 |
| Male | ROL_L | -0.005 (-0.018, 0.008) | 0.481 | 0.923 |
| Male | ROL_R | -0.006 (-0.020, 0.007) | 0.346 | 0.916 |
| Male | SMA_L | -0.001 (-0.011, 0.010) | 0.879 | 0.994 |
| Male | SMA_R | -0.000 (-0.011, 0.010) | 0.961 | 0.994 |
| Male | OLF_L | -0.005 (-0.021, 0.010) | 0.521 | 0.923 |
| Male | OLF_R | -0.000 (-0.016, 0.015) | 0.953 | 0.994 |
| Male | SFGmed_L | 0.004 (-0.003, 0.012) | 0.271 | 0.916 |
| Male | SFGmed_R | 0.009 (-0.001, 0.020) | 0.074 | 0.698 |
| Male | ORBsupmed_L | 0.006 (-0.008, 0.020) | 0.383 | 0.916 |
| Male | ORBsupmed_R | -0.002 (-0.017, 0.013) | 0.811 | 0.994 |
| Male | REC_L | 0.006 (-0.006, 0.018) | 0.307 | 0.916 |
| Male | REC_R | 0.005 (-0.009, 0.020) | 0.48 | 0.923 |
| Male | INS_L | -0.004 (-0.018, 0.011) | 0.606 | 0.94 |
| Male | INS_R | -0.007 (-0.021, 0.006) | 0.291 | 0.916 |
| Male | ACG_L | -0.001 (-0.015, 0.014) | 0.941 | 0.994 |
| Male | ACG_R | 0.002 (-0.014, 0.018) | 0.816 | 0.994 |
| Male | DCG_L | -0.005 (-0.018, 0.007) | 0.414 | 0.916 |
| Male | DCG_R | -0.006 (-0.017, 0.006) | 0.351 | 0.916 |
| Male | PCG_L | -0.008 (-0.025, 0.009) | 0.336 | 0.916 |
| Male | PCG_R | 0.000 (-0.011, 0.011) | 0.983 | 0.994 |
| Male | HIP_L | -0.015 (-0.033, 0.003) | 0.101 | 0.698 |
| Male | HIP_R | -0.016 (-0.031, -0.000) | 0.047 | 0.698 |
| Male | PHG_L | -0.009 (-0.019, 0.001) | 0.088 | 0.698 |
| Male | PHG_R | -0.015 (-0.027, -0.003) | 0.015 | 0.698 |
| Male | AMYG_L | -0.014 (-0.032, 0.004) | 0.13 | 0.779 |
| Male | AMYG_R | -0.006 (-0.023, 0.010) | 0.438 | 0.923 |
| Male | CAL_L | -0.008 (-0.023, 0.007) | 0.287 | 0.916 |
| Male | CAL_R | -0.004 (-0.019, 0.011) | 0.58 | 0.932 |
| Male | CUN_L | 0.003 (-0.009, 0.015) | 0.624 | 0.952 |
| Male | CUN_R | 0.009 (-0.003, 0.021) | 0.139 | 0.782 |
| Male | LING_L | 0.002 (-0.009, 0.013) | 0.712 | 0.971 |
| Male | LING_R | -0.002 (-0.011, 0.008) | 0.732 | 0.971 |
| Male | SOG_L | -0.000 (-0.010, 0.009) | 0.938 | 0.994 |
| Male | SOG_R | 0.000 (-0.010, 0.011) | 0.931 | 0.994 |
| Male | MOG_L | -0.006 (-0.018, 0.006) | 0.31 | 0.916 |
| Male | MOG_R | -0.005 (-0.017, 0.007) | 0.446 | 0.923 |
| Male | IOG_L | -0.012 (-0.026, 0.002) | 0.098 | 0.698 |
| Male | IOG_R | 0.006 (-0.009, 0.021) | 0.417 | 0.916 |
| Male | FFG_L | -0.011 (-0.024, 0.001) | 0.068 | 0.698 |
| Male | FFG_R | -0.007 (-0.018, 0.004) | 0.229 | 0.916 |
| Male | PoCG_L | -0.002 (-0.011, 0.007) | 0.672 | 0.971 |
| Male | PoCG_R | -0.002 (-0.011, 0.007) | 0.665 | 0.971 |
| Male | SPG_L | 0.006 (-0.004, 0.017) | 0.26 | 0.916 |
| Male | SPG_R | 0.009 (-0.000, 0.017) | 0.054 | 0.698 |
| Male | IPL_L | -0.004 (-0.015, 0.008) | 0.547 | 0.929 |
| Male | IPL_R | -0.002 (-0.016, 0.012) | 0.771 | 0.991 |
| Male | SMG_L | -0.015 (-0.030, -0.001) | 0.036 | 0.698 |
| Male | SMG_R | -0.011 (-0.024, 0.001) | 0.082 | 0.698 |
| Male | ANG_L | -0.003 (-0.018, 0.013) | 0.754 | 0.984 |
| Male | ANG_R | -0.004 (-0.017, 0.008) | 0.509 | 0.923 |
| Male | PCUN_L | 0.001 (-0.009, 0.011) | 0.834 | 0.994 |
| Male | PCUN_R | 0.002 (-0.008, 0.012) | 0.734 | 0.971 |
| Male | PCL_L | 0.002 (-0.011, 0.014) | 0.794 | 0.994 |
| Male | PCL_R | 0.005 (-0.009, 0.019) | 0.496 | 0.923 |
| Male | CAU_L | -0.017 (-0.034, 0.000) | 0.054 | 0.698 |
| Male | CAU_R | -0.012 (-0.032, 0.007) | 0.204 | 0.916 |
| Male | PUT_L | -0.011 (-0.035, 0.013) | 0.378 | 0.916 |
| Male | PUT_R | -0.006 (-0.026, 0.015) | 0.574 | 0.932 |
| Male | PAL_L | -0.003 (-0.015, 0.009) | 0.602 | 0.94 |
| Male | PAL_R | -0.000 (-0.015, 0.015) | 0.969 | 0.994 |
| Male | THA_L | -0.008 (-0.032, 0.016) | 0.523 | 0.923 |
| Male | THA_R | -0.011 (-0.034, 0.013) | 0.385 | 0.916 |
| Male | HES_L | -0.008 (-0.028, 0.011) | 0.41 | 0.916 |
| Male | HES_R | -0.009 (-0.028, 0.011) | 0.375 | 0.916 |
| Male | STG_L | -0.012 (-0.024, 0.001) | 0.08 | 0.698 |
| Male | STG_R | 0.002 (-0.007, 0.012) | 0.635 | 0.952 |
| Male | TPOsup_L | 0.004 (-0.005, 0.013) | 0.389 | 0.916 |
| Male | TPOsup_R | -0.000 (-0.010, 0.010) | 0.975 | 0.994 |
| Male | MTG_L | -0.001 (-0.012, 0.010) | 0.843 | 0.994 |
| Male | MTG_R | 0.000 (-0.010, 0.010) | 0.994 | 0.994 |
| Male | TPOmid_L | -0.001 (-0.015, 0.014) | 0.929 | 0.994 |
| Male | TPOmid_R | -0.004 (-0.014, 0.007) | 0.523 | 0.923 |
| Male | ITG_L | -0.001 (-0.012, 0.011) | 0.913 | 0.994 |
| Male | ITG_R | -0.002 (-0.012, 0.008) | 0.717 | 0.971 |
| Female | PreCG_L | -0.004 (-0.012, 0.003) | 0.272 | 0.408 |
| Female | PreCG_R | -0.001 (-0.009, 0.007) | 0.736 | 0.808 |
| Female | SFGdor_L | -0.003 (-0.009, 0.004) | 0.465 | 0.606 |
| Female | SFGdor_R | -0.004 (-0.011, 0.002) | 0.203 | 0.338 |
| Female | ORBsup_L | -0.018 (-0.029, -0.007) | 0.001 | 0.016 |
| Female | ORBsup_R | -0.021 (-0.032, -0.011) | <0.001 | 0.003 |
| Female | MFG_L | -0.006 (-0.013, 0.002) | 0.157 | 0.277 |
| Female | MFG_R | -0.008 (-0.016, -0.000) | 0.047 | 0.129 |
| Female | ORBmid_L | -0.017 (-0.028, -0.007) | 0.001 | 0.016 |
| Female | ORBmid_R | -0.013 (-0.024, -0.003) | 0.011 | 0.063 |
| Female | IFGoperc_L | -0.003 (-0.011, 0.006) | 0.508 | 0.635 |
| Female | IFGoperc_R | -0.005 (-0.015, 0.005) | 0.321 | 0.466 |
| Female | IFGtriang_L | -0.003 (-0.012, 0.006) | 0.476 | 0.612 |
| Female | IFGtriang_R | -0.003 (-0.011, 0.006) | 0.517 | 0.637 |
| Female | ORBinf_L | -0.012 (-0.021, -0.003) | 0.006 | 0.062 |
| Female | ORBinf_R | -0.009 (-0.017, -0.001) | 0.023 | 0.089 |
| Female | ROL_L | -0.011 (-0.021, 0.000) | 0.052 | 0.137 |
| Female | ROL_R | -0.013 (-0.024, -0.003) | 0.015 | 0.069 |
| Female | SMA_L | 0.001 (-0.007, 0.010) | 0.776 | 0.832 |
| Female | SMA_R | 0.001 (-0.007, 0.010) | 0.8 | 0.843 |
| Female | OLF_L | -0.006 (-0.019, 0.007) | 0.398 | 0.535 |
| Female | OLF_R | -0.015 (-0.027, -0.002) | 0.021 | 0.087 |
| Female | SFGmed_L | -0.002 (-0.009, 0.004) | 0.449 | 0.595 |
| Female | SFGmed_R | -0.005 (-0.014, 0.003) | 0.235 | 0.372 |
| Female | ORBsupmed_L | -0.019 (-0.031, -0.008) | 0.001 | 0.016 |
| Female | ORBsupmed_R | -0.017 (-0.030, -0.004) | 0.013 | 0.068 |
| Female | REC_L | -0.012 (-0.022, -0.001) | 0.032 | 0.103 |
| Female | REC_R | -0.014 (-0.026, -0.002) | 0.027 | 0.098 |
| Female | INS_L | -0.003 (-0.015, 0.008) | 0.578 | 0.675 |
| Female | INS_R | -0.011 (-0.023, -0.000) | 0.044 | 0.127 |
| Female | ACG_L | -0.010 (-0.023, 0.002) | 0.113 | 0.231 |
| Female | ACG_R | -0.017 (-0.031, -0.004) | 0.011 | 0.063 |
| Female | DCG_L | -0.005 (-0.015, 0.005) | 0.301 | 0.445 |
| Female | DCG_R | -0.008 (-0.018, 0.001) | 0.083 | 0.187 |
| Female | PCG_L | -0.016 (-0.029, -0.002) | 0.024 | 0.089 |
| Female | PCG_R | -0.009 (-0.018, -0.001) | 0.029 | 0.099 |
| Female | HIP_L | -0.001 (-0.014, 0.012) | 0.839 | 0.868 |
| Female | HIP_R | 0.003 (-0.008, 0.015) | 0.557 | 0.669 |
| Female | PHG_L | 0.010 (0.002, 0.017) | 0.011 | 0.063 |
| Female | PHG_R | 0.000 (-0.009, 0.010) | 0.939 | 0.95 |
| Female | AMYG_L | 0.009 (-0.004, 0.022) | 0.154 | 0.277 |
| Female | AMYG_R | 0.007 (-0.005, 0.019) | 0.231 | 0.371 |
| Female | CAL_L | -0.019 (-0.032, -0.005) | 0.008 | 0.062 |
| Female | CAL_R | -0.014 (-0.027, -0.000) | 0.043 | 0.127 |
| Female | CUN_L | -0.003 (-0.014, 0.008) | 0.565 | 0.669 |
| Female | CUN_R | -0.005 (-0.016, 0.006) | 0.38 | 0.519 |
| Female | LING_L | -0.006 (-0.014, 0.003) | 0.184 | 0.313 |
| Female | LING_R | -0.007 (-0.015, 0.001) | 0.077 | 0.182 |
| Female | SOG_L | -0.007 (-0.015, 0.002) | 0.12 | 0.235 |
| Female | SOG_R | -0.004 (-0.014, 0.005) | 0.355 | 0.5 |
| Female | MOG_L | -0.012 (-0.022, -0.002) | 0.017 | 0.073 |
| Female | MOG_R | -0.007 (-0.017, 0.003) | 0.156 | 0.277 |
| Female | IOG_L | -0.007 (-0.018, 0.005) | 0.261 | 0.398 |
| Female | IOG_R | -0.001 (-0.012, 0.011) | 0.927 | 0.948 |
| Female | FFG_L | 0.002 (-0.009, 0.012) | 0.748 | 0.811 |
| Female | FFG_R | -0.006 (-0.015, 0.003) | 0.177 | 0.306 |
| Female | PoCG_L | -0.007 (-0.014, 0.000) | 0.055 | 0.138 |
| Female | PoCG_R | -0.007 (-0.014, -0.000) | 0.047 | 0.129 |
| Female | SPG_L | -0.008 (-0.017, 0.000) | 0.054 | 0.138 |
| Female | SPG_R | -0.004 (-0.011, 0.003) | 0.255 | 0.396 |
| Female | IPL_L | -0.004 (-0.014, 0.005) | 0.361 | 0.5 |
| Female | IPL_R | -0.002 (-0.014, 0.009) | 0.711 | 0.79 |
| Female | SMG_L | -0.015 (-0.027, -0.003) | 0.014 | 0.069 |
| Female | SMG_R | -0.013 (-0.022, -0.003) | 0.01 | 0.063 |
| Female | ANG_L | -0.010 (-0.023, 0.002) | 0.108 | 0.227 |
| Female | ANG_R | -0.003 (-0.014, 0.008) | 0.589 | 0.679 |
| Female | PCUN_L | -0.012 (-0.020, -0.005) | 0.001 | 0.016 |
| Female | PCUN_R | -0.007 (-0.015, 0.001) | 0.086 | 0.189 |
| Female | PCL_L | 0.002 (-0.008, 0.012) | 0.641 | 0.721 |
| Female | PCL_R | -0.001 (-0.013, 0.010) | 0.805 | 0.843 |
| Female | CAU_L | 0.004 (-0.010, 0.018) | 0.609 | 0.694 |
| Female | CAU_R | 0.013 (-0.003, 0.029) | 0.109 | 0.227 |
| Female | PUT_L | 0.037 (0.019, 0.056) | <0.001 | 0.003 |
| Female | PUT_R | 0.027 (0.011, 0.043) | 0.001 | 0.016 |
| Female | PAL_L | 0.015 (0.003, 0.027) | 0.015 | 0.069 |
| Female | PAL_R | 0.022 (0.009, 0.035) | 0.001 | 0.016 |
| Female | THA_L | -0.023 (-0.040, -0.006) | 0.008 | 0.062 |
| Female | THA_R | -0.023 (-0.040, -0.006) | 0.01 | 0.063 |
| Female | HES_L | -0.016 (-0.032, 0.000) | 0.058 | 0.14 |
| Female | HES_R | -0.015 (-0.032, 0.002) | 0.08 | 0.184 |
| Female | STG_L | -0.011 (-0.022, -0.001) | 0.03 | 0.099 |
| Female | STG_R | -0.006 (-0.014, 0.002) | 0.151 | 0.277 |
| Female | TPOsup_L | -0.004 (-0.012, 0.004) | 0.327 | 0.467 |
| Female | TPOsup_R | -0.006 (-0.015, 0.002) | 0.155 | 0.277 |
| Female | MTG_L | -0.007 (-0.016, 0.002) | 0.117 | 0.234 |
| Female | MTG_R | 0.002 (-0.006, 0.010) | 0.563 | 0.669 |
| Female | TPOmid_L | -0.000 (-0.013, 0.012) | 0.979 | 0.979 |
| Female | TPOmid_R | -0.011 (-0.021, -0.001) | 0.038 | 0.118 |
| Female | ITG_L | -0.003 (-0.012, 0.006) | 0.506 | 0.635 |
| Female | ITG_R | 0.005 (-0.003, 0.014) | 0.231 | 0.371 |

Models were adjusted for age, sex, and total intracranial volume (TIV)

Supplementary Table S5. Associations of cumulative WWI with regional white matter hyperintensity volumes

| Group | Outcome | Beta (95% CI) | *P* | P_FDR_ |
| --- | --- | --- | --- | --- |
| Total | log WMH Frontal lobe L | 0.078 (0.042, 0.115) | <0.001 | <0.001 |
| Total | log WMH Frontal lobe R | 0.074 (0.039, 0.109) | <0.001 | <0.001 |
| Total | log WMH Occipital lobe L | 0.039 (0.015, 0.062) | 0.001 | 0.003 |
| Total | log WMH Occipital lobe R | 0.031 (0.010, 0.052) | 0.004 | 0.005 |
| Total | log WMH Parietal lobe L | 0.026 (0.004, 0.048) | 0.021 | 0.023 |
| Total | log WMH Parietal lobe R | 0.016 (-0.003, 0.034) | 0.095 | 0.095 |
| Total | log WMH Temporal lobe L | 0.048 (0.018, 0.078) | 0.002 | 0.004 |
| Total | log WMH Temporal lobe R | 0.039 (0.012, 0.066) | 0.004 | 0.005 |
| Total | log WMH Limbic lobe L | 0.029 (0.010, 0.048) | 0.003 | 0.005 |
| Total | log WMH Limbic lobe R | 0.026 (0.008, 0.044) | 0.004 | 0.005 |
| Total | log WMH Sub lobe L | 0.057 (0.031, 0.084) | <0.001 | <0.001 |
| Total | log WMH Sub lobe R | 0.052 (0.027, 0.077) | <0.001 | <0.001 |
| Male | log WMH Frontal lobe L | 0.052 (-0.012, 0.116) | 0.109 | 0.404 |
| Male | log WMH Frontal lobe R | 0.058 (-0.004, 0.120) | 0.069 | 0.404 |
| Male | log WMH Occipital lobe L | 0.025 (-0.015, 0.064) | 0.218 | 0.404 |
| Male | log WMH Occipital lobe R | 0.019 (-0.017, 0.056) | 0.303 | 0.404 |
| Male | log WMH Parietal lobe L | 0.023 (-0.018, 0.064) | 0.276 | 0.404 |
| Male | log WMH Parietal lobe R | 0.015 (-0.019, 0.050) | 0.382 | 0.458 |
| Male | log WMH Temporal lobe L | 0.033 (-0.019, 0.085) | 0.218 | 0.404 |
| Male | log WMH Temporal lobe R | 0.031 (-0.015, 0.077) | 0.183 | 0.404 |
| Male | log WMH Limbic lobe L | 0.013 (-0.021, 0.047) | 0.466 | 0.509 |
| Male | log WMH Limbic lobe R | 0.007 (-0.025, 0.039) | 0.676 | 0.676 |
| Male | log WMH Sub lobe L | 0.031 (-0.013, 0.074) | 0.165 | 0.404 |
| Male | log WMH Sub lobe R | 0.024 (-0.016, 0.065) | 0.243 | 0.404 |
| Female | log WMH Frontal lobe L | 0.107 (0.070, 0.144) | <0.001 | <0.001 |
| Female | log WMH Frontal lobe R | 0.097 (0.061, 0.132) | <0.001 | <0.001 |
| Female | log WMH Occipital lobe L | 0.054 (0.027, 0.081) | <0.001 | <0.001 |
| Female | log WMH Occipital lobe R | 0.046 (0.023, 0.068) | <0.001 | <0.001 |
| Female | log WMH Parietal lobe L | 0.035 (0.016, 0.054) | <0.001 | <0.001 |
| Female | log WMH Parietal lobe R | 0.022 (0.008, 0.037) | 0.002 | 0.002 |
| Female | log WMH Temporal lobe L | 0.066 (0.033, 0.099) | <0.001 | <0.001 |
| Female | log WMH Temporal lobe R | 0.052 (0.023, 0.080) | <0.001 | <0.001 |
| Female | log WMH Limbic lobe L | 0.045 (0.027, 0.063) | <0.001 | <0.001 |
| Female | log WMH Limbic lobe R | 0.046 (0.029, 0.062) | <0.001 | <0.001 |
| Female | log WMH Sub lobe L | 0.079 (0.047, 0.111) | <0.001 | <0.001 |
| Female | log WMH Sub lobe R | 0.075 (0.046, 0.105) | <0.001 | <0.001 |

Models were adjusted for age, sex, and total intracranial volume (TIV)

Supplementary Table S6. Associations of cumulative WWI with regional diffusion tensor imaging metrics (FA and MD)

| Group | Outcome | Beta (95% CI) | *P* | P_FDR_ |
| --- | --- | --- | --- | --- |
| Total | FA Middle cerebellar peduncle | 0.019 (-0.045, 0.083) | 0.563 | 0.807 |
| Total | FA Pontine crossing tract a part of MCP | 0.044 (-0.023, 0.112) | 0.199 | 0.503 |
| Total | FA Genu of corpus callosum | 0.004 (-0.054, 0.061) | 0.902 | 0.941 |
| Total | FA Body of corpus callosum | 0.010 (-0.050, 0.070) | 0.74 | 0.866 |
| Total | FA Splenium of corpus callosum | 0.026 (-0.037, 0.090) | 0.415 | 0.712 |
| Total | FA Fornix column and body of fornix | -0.068 (-0.127, -0.010) | 0.022 | 0.233 |
| Total | FA Corticospinal tract R | 0.035 (-0.032, 0.103) | 0.305 | 0.655 |
| Total | FA Corticospinal tract L | 0.075 (0.008, 0.143) | 0.029 | 0.233 |
| Total | FA Medial lemniscus R | -0.021 (-0.089, 0.048) | 0.553 | 0.805 |
| Total | FA Medial lemniscus L | -0.055 (-0.124, 0.013) | 0.115 | 0.379 |
| Total | FA Inferior cerebellar peduncle R | -0.013 (-0.078, 0.051) | 0.684 | 0.832 |
| Total | FA Inferior cerebellar peduncle L | -0.023 (-0.089, 0.043) | 0.497 | 0.789 |
| Total | FA Superior cerebellar peduncle R | -0.015 (-0.084, 0.054) | 0.666 | 0.832 |
| Total | FA Superior cerebellar peduncle L | -0.013 (-0.082, 0.056) | 0.712 | 0.844 |
| Total | FA Cerebral peduncle R | -0.007 (-0.075, 0.061) | 0.838 | 0.904 |
| Total | FA Cerebral peduncle L | 0.016 (-0.050, 0.082) | 0.639 | 0.832 |
| Total | FA Anterior limb of internal capsule R | -0.029 (-0.093, 0.035) | 0.371 | 0.712 |
| Total | FA Anterior limb of internal capsule L | -0.009 (-0.072, 0.054) | 0.777 | 0.888 |
| Total | FA Posterior limb of internal capsule R | 0.020 (-0.045, 0.084) | 0.548 | 0.805 |
| Total | FA Posterior limb of internal capsule L | 0.022 (-0.043, 0.087) | 0.511 | 0.789 |
| Total | FA Retrolenticular part of internal capsule R | 0.018 (-0.050, 0.087) | 0.6 | 0.812 |
| Total | FA Retrolenticular part of internal capsule L | 0.003 (-0.065, 0.071) | 0.939 | 0.949 |
| Total | FA Anterior corona radiata R | -0.035 (-0.098, 0.028) | 0.28 | 0.641 |
| Total | FA Anterior corona radiata L | -0.023 (-0.082, 0.036) | 0.451 | 0.76 |
| Total | FA Superior corona radiata R | -0.008 (-0.072, 0.056) | 0.805 | 0.888 |
| Total | FA Superior corona radiata L | -0.003 (-0.065, 0.059) | 0.919 | 0.949 |
| Total | FA Posterior corona radiata R | 0.028 (-0.036, 0.093) | 0.387 | 0.712 |
| Total | FA Posterior corona radiata L | 0.031 (-0.033, 0.095) | 0.337 | 0.702 |
| Total | FA Posterior thalamic radiation include optic radiation R | -0.015 (-0.082, 0.051) | 0.656 | 0.832 |
| Total | FA Posterior thalamic radiation include optic radiation L | -0.008 (-0.074, 0.059) | 0.815 | 0.889 |
| Total | FA Sagittal stratum include inferior longitidinal fasciculus and inferior fronto occipital fasciculus R | -0.014 (-0.079, 0.050) | 0.66 | 0.832 |
| Total | FA Sagittal stratum include inferior longitidinal fasciculus and inferior fronto occipital fasciculus L | -0.027 (-0.090, 0.036) | 0.405 | 0.712 |
| Total | FA External capsule R | -0.009 (-0.075, 0.057) | 0.8 | 0.888 |
| Total | FA External capsule L | 0.008 (-0.057, 0.074) | 0.8 | 0.888 |
| Total | FA Cingulum cingulate gyrus R | -0.021 (-0.087, 0.044) | 0.526 | 0.789 |
| Total | FA Cingulum cingulate gyrus L | 0.005 (-0.059, 0.069) | 0.873 | 0.931 |
| Total | FA Cingulum hippocampus R | -0.013 (-0.080, 0.055) | 0.711 | 0.844 |
| Total | FA Cingulum hippocampus L | 0.022 (-0.045, 0.090) | 0.519 | 0.789 |
| Total | FA Fornix cres Stria terminalis can not be resolved with current resolution R | -0.024 (-0.082, 0.034) | 0.415 | 0.712 |
| Total | FA Fornix cres Stria terminalis can not be resolved with current resolution L | -0.034 (-0.094, 0.026) | 0.269 | 0.629 |
| Total | FA Superior longitudinal fasciculus R | 0.019 (-0.050, 0.088) | 0.589 | 0.812 |
| Total | FA Superior longitudinal fasciculus L | -0.000 (-0.070, 0.070) | 0.997 | 0.997 |
| Total | FA Superior fronto occipital fasciculus could be a part of anterior internal capsule R | -0.052 (-0.112, 0.009) | 0.095 | 0.35 |
| Total | FA Superior fronto occipital fasciculus could be a part of anterior internal capsule L | -0.063 (-0.124, -0.003) | 0.041 | 0.233 |
| Total | FA Uncinate fasciculus R | 0.009 (-0.060, 0.078) | 0.796 | 0.888 |
| Total | FA Uncinate fasciculus L | 0.060 (-0.008, 0.128) | 0.086 | 0.332 |
| Total | FA Tapetum R | -0.032 (-0.093, 0.029) | 0.307 | 0.655 |
| Total | FA Tapetum L | -0.018 (-0.082, 0.046) | 0.585 | 0.812 |
| Total | MD Middle cerebellar peduncle | 0.044 (-0.023, 0.112) | 0.195 | 0.503 |
| Total | MD Pontine crossing tract a part of MCP | -0.047 (-0.116, 0.022) | 0.183 | 0.488 |
| Total | MD Genu of corpus callosum | 0.054 (-0.002, 0.110) | 0.061 | 0.288 |
| Total | MD Body of corpus callosum | 0.039 (-0.018, 0.096) | 0.182 | 0.488 |
| Total | MD Splenium of corpus callosum | 0.054 (-0.003, 0.112) | 0.063 | 0.288 |
| Total | MD Fornix column and body of fornix | 0.070 (0.017, 0.124) | 0.01 | 0.191 |
| Total | MD Corticospinal tract R | 0.028 (-0.036, 0.092) | 0.389 | 0.712 |
| Total | MD Corticospinal tract L | 0.016 (-0.048, 0.079) | 0.625 | 0.832 |
| Total | MD Medial lemniscus R | 0.025 (-0.045, 0.095) | 0.483 | 0.789 |
| Total | MD Medial lemniscus L | 0.081 (0.011, 0.150) | 0.023 | 0.233 |
| Total | MD Inferior cerebellar peduncle R | 0.014 (-0.055, 0.084) | 0.683 | 0.832 |
| Total | MD Inferior cerebellar peduncle L | 0.023 (-0.046, 0.091) | 0.512 | 0.789 |
| Total | MD Superior cerebellar peduncle R | 0.031 (-0.039, 0.101) | 0.39 | 0.712 |
| Total | MD Superior cerebellar peduncle L | 0.029 (-0.041, 0.099) | 0.414 | 0.712 |
| Total | MD Cerebral peduncle R | 0.024 (-0.045, 0.093) | 0.494 | 0.789 |
| Total | MD Cerebral peduncle L | 0.033 (-0.037, 0.102) | 0.355 | 0.712 |
| Total | MD Anterior limb of internal capsule R | 0.037 (-0.028, 0.101) | 0.263 | 0.629 |
| Total | MD Anterior limb of internal capsule L | 0.064 (0.002, 0.125) | 0.044 | 0.234 |
| Total | MD Posterior limb of internal capsule R | 0.005 (-0.062, 0.072) | 0.886 | 0.934 |
| Total | MD Posterior limb of internal capsule L | 0.081 (0.017, 0.144) | 0.013 | 0.205 |
| Total | MD Retrolenticular part of internal capsule R | 0.043 (-0.015, 0.100) | 0.146 | 0.439 |
| Total | MD Retrolenticular part of internal capsule L | 0.076 (0.022, 0.130) | 0.006 | 0.174 |
| Total | MD Anterior corona radiata R | 0.046 (-0.012, 0.105) | 0.12 | 0.384 |
| Total | MD Anterior corona radiata L | 0.053 (-0.005, 0.110) | 0.073 | 0.305 |
| Total | MD Superior corona radiata R | 0.048 (-0.010, 0.106) | 0.107 | 0.367 |
| Total | MD Superior corona radiata L | 0.078 (0.022, 0.133) | 0.006 | 0.174 |
| Total | MD Posterior corona radiata R | 0.016 (-0.043, 0.074) | 0.593 | 0.812 |
| Total | MD Posterior corona radiata L | 0.031 (-0.026, 0.088) | 0.29 | 0.648 |
| Total | MD Posterior thalamic radiation include optic radiation R | 0.046 (-0.015, 0.107) | 0.141 | 0.438 |
| Total | MD Posterior thalamic radiation include optic radiation L | 0.062 (0.003, 0.122) | 0.041 | 0.233 |
| Total | MD Sagittal stratum include inferior longitidinal fasciculus and inferior fronto occipital fasciculus R | 0.076 (0.021, 0.132) | 0.007 | 0.174 |
| Total | MD Sagittal stratum include inferior longitidinal fasciculus and inferior fronto occipital fasciculus L | 0.054 (-0.003, 0.111) | 0.062 | 0.288 |
| Total | MD External capsule R | 0.053 (-0.006, 0.113) | 0.079 | 0.316 |
| Total | MD External capsule L | 0.066 (0.004, 0.129) | 0.039 | 0.233 |
| Total | MD Cingulum cingulate gyrus R | 0.043 (-0.018, 0.103) | 0.169 | 0.477 |
| Total | MD Cingulum cingulate gyrus L | 0.033 (-0.025, 0.092) | 0.266 | 0.629 |
| Total | MD Cingulum hippocampus R | 0.063 (0.005, 0.122) | 0.035 | 0.233 |
| Total | MD Cingulum hippocampus L | 0.049 (-0.009, 0.107) | 0.099 | 0.351 |
| Total | MD Fornix cres Stria terminalis can not be resolved with current resolution R | 0.041 (-0.016, 0.097) | 0.16 | 0.465 |
| Total | MD Fornix cres Stria terminalis can not be resolved with current resolution L | 0.063 (0.006, 0.120) | 0.03 | 0.233 |
| Total | MD Superior longitudinal fasciculus R | 0.013 (-0.048, 0.075) | 0.673 | 0.832 |
| Total | MD Superior longitudinal fasciculus L | 0.064 (0.003, 0.125) | 0.04 | 0.233 |
| Total | MD Superior fronto occipital fasciculus could be a part of anterior internal capsule R | 0.065 (0.007, 0.124) | 0.029 | 0.233 |
| Total | MD Superior fronto occipital fasciculus could be a part of anterior internal capsule L | 0.071 (0.012, 0.131) | 0.019 | 0.233 |
| Total | MD Uncinate fasciculus R | 0.003 (-0.064, 0.069) | 0.933 | 0.949 |
| Total | MD Uncinate fasciculus L | -0.031 (-0.100, 0.038) | 0.381 | 0.712 |
| Total | MD Tapetum R | 0.090 (0.030, 0.151) | 0.004 | 0.174 |
| Total | MD Tapetum L | 0.058 (-0.004, 0.120) | 0.068 | 0.298 |
| Male | FA Middle cerebellar peduncle | 0.015 (-0.104, 0.134) | 0.802 | 0.974 |
| Male | FA Pontine crossing tract a part of MCP | 0.057 (-0.061, 0.175) | 0.343 | 0.908 |
| Male | FA Genu of corpus callosum | 0.010 (-0.094, 0.115) | 0.847 | 0.974 |
| Male | FA Body of corpus callosum | 0.029 (-0.074, 0.133) | 0.577 | 0.908 |
| Male | FA Splenium of corpus callosum | 0.044 (-0.073, 0.160) | 0.462 | 0.908 |
| Male | FA Fornix column and body of fornix | -0.133 (-0.228, -0.039) | 0.006 | 0.401 |
| Male | FA Corticospinal tract R | 0.050 (-0.067, 0.168) | 0.398 | 0.908 |
| Male | FA Corticospinal tract L | 0.077 (-0.042, 0.196) | 0.207 | 0.908 |
| Male | FA Medial lemniscus R | -0.029 (-0.156, 0.097) | 0.651 | 0.933 |
| Male | FA Medial lemniscus L | -0.078 (-0.200, 0.043) | 0.208 | 0.908 |
| Male | FA Inferior cerebellar peduncle R | -0.034 (-0.150, 0.081) | 0.559 | 0.908 |
| Male | FA Inferior cerebellar peduncle L | -0.064 (-0.181, 0.054) | 0.288 | 0.908 |
| Male | FA Superior cerebellar peduncle R | -0.002 (-0.130, 0.125) | 0.97 | 0.984 |
| Male | FA Superior cerebellar peduncle L | 0.007 (-0.121, 0.136) | 0.91 | 0.984 |
| Male | FA Cerebral peduncle R | 0.015 (-0.112, 0.143) | 0.815 | 0.974 |
| Male | FA Cerebral peduncle L | 0.047 (-0.072, 0.166) | 0.436 | 0.908 |
| Male | FA Anterior limb of internal capsule R | -0.029 (-0.144, 0.086) | 0.622 | 0.908 |
| Male | FA Anterior limb of internal capsule L | 0.003 (-0.110, 0.116) | 0.962 | 0.984 |
| Male | FA Posterior limb of internal capsule R | 0.061 (-0.058, 0.179) | 0.318 | 0.908 |
| Male | FA Posterior limb of internal capsule L | 0.068 (-0.054, 0.189) | 0.276 | 0.908 |
| Male | FA Retrolenticular part of internal capsule R | 0.068 (-0.047, 0.183) | 0.249 | 0.908 |
| Male | FA Retrolenticular part of internal capsule L | 0.043 (-0.079, 0.166) | 0.49 | 0.908 |
| Male | FA Anterior corona radiata R | -0.036 (-0.151, 0.078) | 0.535 | 0.908 |
| Male | FA Anterior corona radiata L | -0.025 (-0.122, 0.073) | 0.622 | 0.908 |
| Male | FA Superior corona radiata R | 0.022 (-0.095, 0.139) | 0.717 | 0.969 |
| Male | FA Superior corona radiata L | 0.015 (-0.096, 0.126) | 0.787 | 0.974 |
| Male | FA Posterior corona radiata R | 0.082 (-0.034, 0.197) | 0.166 | 0.908 |
| Male | FA Posterior corona radiata L | 0.103 (-0.009, 0.215) | 0.072 | 0.772 |
| Male | FA Posterior thalamic radiation include optic radiation R | 0.033 (-0.080, 0.146) | 0.571 | 0.908 |
| Male | FA Posterior thalamic radiation include optic radiation L | 0.034 (-0.082, 0.150) | 0.566 | 0.908 |
| Male | FA Sagittal stratum include inferior longitidinal fasciculus and inferior fronto occipital fasciculus R | 0.010 (-0.103, 0.122) | 0.868 | 0.98 |
| Male | FA Sagittal stratum include inferior longitidinal fasciculus and inferior fronto occipital fasciculus L | -0.012 (-0.120, 0.096) | 0.828 | 0.974 |
| Male | FA External capsule R | -0.004 (-0.124, 0.116) | 0.945 | 0.984 |
| Male | FA External capsule L | 0.030 (-0.088, 0.149) | 0.614 | 0.908 |
| Male | FA Cingulum cingulate gyrus R | -0.034 (-0.152, 0.084) | 0.576 | 0.908 |
| Male | FA Cingulum cingulate gyrus L | 0.028 (-0.083, 0.139) | 0.622 | 0.908 |
| Male | FA Cingulum hippocampus R | -0.049 (-0.158, 0.061) | 0.382 | 0.908 |
| Male | FA Cingulum hippocampus L | 0.006 (-0.107, 0.120) | 0.912 | 0.984 |
| Male | FA Fornix cres Stria terminalis can not be resolved with current resolution R | -0.039 (-0.140, 0.061) | 0.445 | 0.908 |
| Male | FA Fornix cres Stria terminalis can not be resolved with current resolution L | -0.046 (-0.154, 0.061) | 0.401 | 0.908 |
| Male | FA Superior longitudinal fasciculus R | 0.069 (-0.053, 0.190) | 0.267 | 0.908 |
| Male | FA Superior longitudinal fasciculus L | 0.044 (-0.079, 0.168) | 0.483 | 0.908 |
| Male | FA Superior fronto occipital fasciculus could be a part of anterior internal capsule R | -0.071 (-0.176, 0.033) | 0.181 | 0.908 |
| Male | FA Superior fronto occipital fasciculus could be a part of anterior internal capsule L | -0.130 (-0.234, -0.027) | 0.014 | 0.444 |
| Male | FA Uncinate fasciculus R | 0.004 (-0.116, 0.124) | 0.953 | 0.984 |
| Male | FA Uncinate fasciculus L | 0.066 (-0.049, 0.182) | 0.262 | 0.908 |
| Male | FA Tapetum R | -0.056 (-0.170, 0.058) | 0.334 | 0.908 |
| Male | FA Tapetum L | -0.030 (-0.148, 0.089) | 0.624 | 0.908 |
| Male | MD Middle cerebellar peduncle | 0.033 (-0.082, 0.149) | 0.569 | 0.908 |
| Male | MD Pontine crossing tract a part of MCP | -0.061 (-0.190, 0.068) | 0.355 | 0.908 |
| Male | MD Genu of corpus callosum | 0.104 (0.008, 0.200) | 0.034 | 0.552 |
| Male | MD Body of corpus callosum | 0.040 (-0.056, 0.137) | 0.415 | 0.908 |
| Male | MD Splenium of corpus callosum | 0.033 (-0.068, 0.133) | 0.525 | 0.908 |
| Male | MD Fornix column and body of fornix | 0.115 (0.030, 0.200) | 0.008 | 0.401 |
| Male | MD Corticospinal tract R | 0.014 (-0.097, 0.125) | 0.805 | 0.974 |
| Male | MD Corticospinal tract L | -0.023 (-0.131, 0.084) | 0.671 | 0.947 |
| Male | MD Medial lemniscus R | -0.017 (-0.151, 0.118) | 0.809 | 0.974 |
| Male | MD Medial lemniscus L | 0.059 (-0.072, 0.190) | 0.379 | 0.908 |
| Male | MD Inferior cerebellar peduncle R | -0.014 (-0.132, 0.104) | 0.818 | 0.974 |
| Male | MD Inferior cerebellar peduncle L | 0.045 (-0.074, 0.164) | 0.457 | 0.908 |
| Male | MD Superior cerebellar peduncle R | -0.006 (-0.123, 0.112) | 0.924 | 0.984 |
| Male | MD Superior cerebellar peduncle L | 0.014 (-0.106, 0.134) | 0.818 | 0.974 |
| Male | MD Cerebral peduncle R | 0.002 (-0.124, 0.127) | 0.979 | 0.984 |
| Male | MD Cerebral peduncle L | 0.046 (-0.074, 0.166) | 0.452 | 0.908 |
| Male | MD Anterior limb of internal capsule R | -0.013 (-0.130, 0.104) | 0.828 | 0.974 |
| Male | MD Anterior limb of internal capsule L | 0.033 (-0.072, 0.139) | 0.534 | 0.908 |
| Male | MD Posterior limb of internal capsule R | -0.035 (-0.165, 0.095) | 0.598 | 0.908 |
| Male | MD Posterior limb of internal capsule L | 0.066 (-0.050, 0.182) | 0.265 | 0.908 |
| Male | MD Retrolenticular part of internal capsule R | 0.021 (-0.083, 0.126) | 0.688 | 0.958 |
| Male | MD Retrolenticular part of internal capsule L | 0.060 (-0.038, 0.158) | 0.228 | 0.908 |
| Male | MD Anterior corona radiata R | 0.052 (-0.057, 0.161) | 0.35 | 0.908 |
| Male | MD Anterior corona radiata L | 0.041 (-0.065, 0.147) | 0.45 | 0.908 |
| Male | MD Superior corona radiata R | 0.047 (-0.060, 0.154) | 0.394 | 0.908 |
| Male | MD Superior corona radiata L | 0.112 (0.010, 0.214) | 0.031 | 0.552 |
| Male | MD Posterior corona radiata R | 0.011 (-0.094, 0.117) | 0.834 | 0.974 |
| Male | MD Posterior corona radiata L | 0.015 (-0.085, 0.114) | 0.774 | 0.974 |
| Male | MD Posterior thalamic radiation include optic radiation R | -0.037 (-0.139, 0.065) | 0.474 | 0.908 |
| Male | MD Posterior thalamic radiation include optic radiation L | 0.033 (-0.064, 0.130) | 0.502 | 0.908 |
| Male | MD Sagittal stratum include inferior longitidinal fasciculus and inferior fronto occipital fasciculus R | 0.057 (-0.039, 0.153) | 0.242 | 0.908 |
| Male | MD Sagittal stratum include inferior longitidinal fasciculus and inferior fronto occipital fasciculus L | 0.061 (-0.038, 0.160) | 0.228 | 0.908 |
| Male | MD External capsule R | 0.005 (-0.102, 0.112) | 0.925 | 0.984 |
| Male | MD External capsule L | 0.052 (-0.069, 0.173) | 0.402 | 0.908 |
| Male | MD Cingulum cingulate gyrus R | 0.006 (-0.098, 0.110) | 0.904 | 0.984 |
| Male | MD Cingulum cingulate gyrus L | -0.001 (-0.097, 0.095) | 0.984 | 0.984 |
| Male | MD Cingulum hippocampus R | 0.042 (-0.065, 0.148) | 0.443 | 0.908 |
| Male | MD Cingulum hippocampus L | 0.019 (-0.082, 0.120) | 0.711 | 0.969 |
| Male | MD Fornix cres Stria terminalis can not be resolved with current resolution R | 0.054 (-0.048, 0.156) | 0.298 | 0.908 |
| Male | MD Fornix cres Stria terminalis can not be resolved with current resolution L | 0.102 (-0.003, 0.206) | 0.057 | 0.683 |
| Male | MD Superior longitudinal fasciculus R | -0.068 (-0.172, 0.035) | 0.197 | 0.908 |
| Male | MD Superior longitudinal fasciculus L | 0.010 (-0.099, 0.119) | 0.852 | 0.974 |
| Male | MD Superior fronto occipital fasciculus could be a part of anterior internal capsule R | 0.080 (-0.030, 0.190) | 0.154 | 0.908 |
| Male | MD Superior fronto occipital fasciculus could be a part of anterior internal capsule L | 0.115 (0.002, 0.227) | 0.046 | 0.632 |
| Male | MD Uncinate fasciculus R | -0.073 (-0.188, 0.041) | 0.211 | 0.908 |
| Male | MD Uncinate fasciculus L | -0.035 (-0.160, 0.091) | 0.587 | 0.908 |
| Male | MD Tapetum R | 0.112 (0.017, 0.206) | 0.021 | 0.496 |
| Male | MD Tapetum L | 0.043 (-0.050, 0.135) | 0.365 | 0.908 |
| Female | FA Middle cerebellar peduncle | 0.009 (-0.046, 0.064) | 0.749 | 0.799 |
| Female | FA Pontine crossing tract a part of MCP | 0.026 (-0.046, 0.098) | 0.479 | 0.64 |
| Female | FA Genu of corpus callosum | -0.012 (-0.067, 0.042) | 0.659 | 0.719 |
| Female | FA Body of corpus callosum | -0.015 (-0.079, 0.050) | 0.654 | 0.719 |
| Female | FA Splenium of corpus callosum | -0.000 (-0.058, 0.058) | 0.994 | 0.994 |
| Female | FA Fornix column and body of fornix | -0.028 (-0.100, 0.043) | 0.433 | 0.63 |
| Female | FA Corticospinal tract R | 0.013 (-0.059, 0.085) | 0.727 | 0.784 |
| Female | FA Corticospinal tract L | 0.060 (-0.010, 0.129) | 0.094 | 0.28 |
| Female | FA Medial lemniscus R | -0.020 (-0.081, 0.042) | 0.531 | 0.655 |
| Female | FA Medial lemniscus L | -0.043 (-0.114, 0.028) | 0.234 | 0.431 |
| Female | FA Inferior cerebellar peduncle R | -0.008 (-0.074, 0.057) | 0.803 | 0.847 |
| Female | FA Inferior cerebellar peduncle L | -0.001 (-0.066, 0.065) | 0.988 | 0.994 |
| Female | FA Superior cerebellar peduncle R | -0.040 (-0.100, 0.021) | 0.201 | 0.405 |
| Female | FA Superior cerebellar peduncle L | -0.037 (-0.097, 0.023) | 0.228 | 0.431 |
| Female | FA Cerebral peduncle R | -0.028 (-0.084, 0.029) | 0.335 | 0.555 |
| Female | FA Cerebral peduncle L | -0.016 (-0.080, 0.047) | 0.612 | 0.689 |
| Female | FA Anterior limb of internal capsule R | -0.037 (-0.100, 0.025) | 0.243 | 0.439 |
| Female | FA Anterior limb of internal capsule L | -0.027 (-0.090, 0.037) | 0.411 | 0.623 |
| Female | FA Posterior limb of internal capsule R | -0.018 (-0.078, 0.041) | 0.542 | 0.655 |
| Female | FA Posterior limb of internal capsule L | -0.016 (-0.074, 0.042) | 0.59 | 0.675 |
| Female | FA Retrolenticular part of internal capsule R | -0.031 (-0.109, 0.046) | 0.431 | 0.63 |
| Female | FA Retrolenticular part of internal capsule L | -0.038 (-0.104, 0.028) | 0.256 | 0.454 |
| Female | FA Anterior corona radiata R | -0.046 (-0.106, 0.013) | 0.128 | 0.341 |
| Female | FA Anterior corona radiata L | -0.037 (-0.105, 0.031) | 0.282 | 0.476 |
| Female | FA Superior corona radiata R | -0.036 (-0.095, 0.022) | 0.22 | 0.431 |
| Female | FA Superior corona radiata L | -0.023 (-0.085, 0.038) | 0.461 | 0.64 |
| Female | FA Posterior corona radiata R | -0.020 (-0.083, 0.042) | 0.525 | 0.655 |
| Female | FA Posterior corona radiata L | -0.029 (-0.096, 0.038) | 0.396 | 0.623 |
| Female | FA Posterior thalamic radiation include optic radiation R | -0.063 (-0.137, 0.011) | 0.097 | 0.28 |
| Female | FA Posterior thalamic radiation include optic radiation L | -0.048 (-0.119, 0.023) | 0.183 | 0.391 |
| Female | FA Sagittal stratum include inferior longitidinal fasciculus and inferior fronto occipital fasciculus R | -0.044 (-0.112, 0.023) | 0.201 | 0.405 |
| Female | FA Sagittal stratum include inferior longitidinal fasciculus and inferior fronto occipital fasciculus L | -0.052 (-0.120, 0.016) | 0.133 | 0.345 |
| Female | FA External capsule R | -0.026 (-0.087, 0.036) | 0.413 | 0.623 |
| Female | FA External capsule L | -0.018 (-0.081, 0.044) | 0.567 | 0.665 |
| Female | FA Cingulum cingulate gyrus R | -0.020 (-0.084, 0.044) | 0.546 | 0.655 |
| Female | FA Cingulum cingulate gyrus L | -0.023 (-0.092, 0.045) | 0.503 | 0.644 |
| Female | FA Cingulum hippocampus R | 0.004 (-0.077, 0.086) | 0.917 | 0.947 |
| Female | FA Cingulum hippocampus L | 0.023 (-0.055, 0.100) | 0.568 | 0.665 |
| Female | FA Fornix cres Stria terminalis can not be resolved with current resolution R | -0.026 (-0.087, 0.036) | 0.415 | 0.623 |
| Female | FA Fornix cres Stria terminalis can not be resolved with current resolution L | -0.038 (-0.096, 0.020) | 0.202 | 0.405 |
| Female | FA Superior longitudinal fasciculus R | -0.021 (-0.092, 0.051) | 0.575 | 0.665 |
| Female | FA Superior longitudinal fasciculus L | -0.033 (-0.104, 0.039) | 0.375 | 0.609 |
| Female | FA Superior fronto occipital fasciculus could be a part of anterior internal capsule R | -0.049 (-0.114, 0.016) | 0.142 | 0.36 |
| Female | FA Superior fronto occipital fasciculus could be a part of anterior internal capsule L | -0.025 (-0.093, 0.042) | 0.458 | 0.64 |
| Female | FA Uncinate fasciculus R | 0.006 (-0.070, 0.081) | 0.881 | 0.919 |
| Female | FA Uncinate fasciculus L | 0.048 (-0.030, 0.125) | 0.231 | 0.431 |
| Female | FA Tapetum R | -0.020 (-0.074, 0.035) | 0.48 | 0.64 |
| Female | FA Tapetum L | -0.015 (-0.075, 0.044) | 0.617 | 0.689 |
| Female | MD Middle cerebellar peduncle | 0.054 (-0.021, 0.129) | 0.158 | 0.378 |
| Female | MD Pontine crossing tract a part of MCP | -0.026 (-0.086, 0.033) | 0.381 | 0.61 |
| Female | MD Genu of corpus callosum | 0.020 (-0.043, 0.083) | 0.534 | 0.655 |
| Female | MD Body of corpus callosum | 0.045 (-0.019, 0.109) | 0.172 | 0.391 |
| Female | MD Splenium of corpus callosum | 0.083 (0.024, 0.142) | 0.006 | 0.039 |
| Female | MD Fornix column and body of fornix | 0.045 (-0.021, 0.111) | 0.18 | 0.391 |
| Female | MD Corticospinal tract R | 0.040 (-0.031, 0.110) | 0.269 | 0.462 |
| Female | MD Corticospinal tract L | 0.050 (-0.022, 0.123) | 0.174 | 0.391 |
| Female | MD Medial lemniscus R | 0.062 (0.009, 0.115) | 0.023 | 0.096 |
| Female | MD Medial lemniscus L | 0.103 (0.045, 0.161) | <0.001 | 0.008 |
| Female | MD Inferior cerebellar peduncle R | 0.029 (-0.050, 0.107) | 0.475 | 0.64 |
| Female | MD Inferior cerebellar peduncle L | 0.003 (-0.071, 0.077) | 0.941 | 0.962 |
| Female | MD Superior cerebellar peduncle R | 0.046 (-0.034, 0.127) | 0.262 | 0.457 |
| Female | MD Superior cerebellar peduncle L | 0.027 (-0.050, 0.104) | 0.493 | 0.644 |
| Female | MD Cerebral peduncle R | 0.047 (-0.018, 0.112) | 0.155 | 0.378 |
| Female | MD Cerebral peduncle L | 0.027 (-0.048, 0.102) | 0.48 | 0.64 |
| Female | MD Anterior limb of internal capsule R | 0.089 (0.032, 0.147) | 0.003 | 0.021 |
| Female | MD Anterior limb of internal capsule L | 0.095 (0.026, 0.164) | 0.007 | 0.041 |
| Female | MD Posterior limb of internal capsule R | 0.049 (0.002, 0.095) | 0.04 | 0.135 |
| Female | MD Posterior limb of internal capsule L | 0.103 (0.045, 0.161) | <0.001 | 0.008 |
| Female | MD Retrolenticular part of internal capsule R | 0.075 (0.024, 0.127) | 0.005 | 0.031 |
| Female | MD Retrolenticular part of internal capsule L | 0.105 (0.055, 0.154) | <0.001 | 0.004 |
| Female | MD Anterior corona radiata R | 0.056 (0.007, 0.105) | 0.025 | 0.098 |
| Female | MD Anterior corona radiata L | 0.076 (0.027, 0.125) | 0.003 | 0.021 |
| Female | MD Superior corona radiata R | 0.068 (0.019, 0.117) | 0.007 | 0.041 |
| Female | MD Superior corona radiata L | 0.069 (0.022, 0.116) | 0.004 | 0.03 |
| Female | MD Posterior corona radiata R | 0.037 (-0.017, 0.091) | 0.183 | 0.391 |
| Female | MD Posterior corona radiata L | 0.060 (0.003, 0.118) | 0.041 | 0.135 |
| Female | MD Posterior thalamic radiation include optic radiation R | 0.120 (0.049, 0.192) | 0.001 | 0.011 |
| Female | MD Posterior thalamic radiation include optic radiation L | 0.091 (0.019, 0.164) | 0.014 | 0.065 |
| Female | MD Sagittal stratum include inferior longitidinal fasciculus and inferior fronto occipital fasciculus R | 0.106 (0.048, 0.164) | <0.001 | 0.007 |
| Female | MD Sagittal stratum include inferior longitidinal fasciculus and inferior fronto occipital fasciculus L | 0.062 (0.004, 0.120) | 0.038 | 0.135 |
| Female | MD External capsule R | 0.107 (0.052, 0.162) | <0.001 | 0.004 |
| Female | MD External capsule L | 0.089 (0.045, 0.133) | <0.001 | 0.004 |
| Female | MD Cingulum cingulate gyrus R | 0.084 (0.018, 0.150) | 0.013 | 0.064 |
| Female | MD Cingulum cingulate gyrus L | 0.069 (0.001, 0.138) | 0.048 | 0.155 |
| Female | MD Cingulum hippocampus R | 0.094 (0.038, 0.149) | <0.001 | 0.011 |
| Female | MD Cingulum hippocampus L | 0.084 (0.022, 0.146) | 0.008 | 0.044 |
| Female | MD Fornix cres Stria terminalis can not be resolved with current resolution R | 0.044 (-0.009, 0.097) | 0.102 | 0.28 |
| Female | MD Fornix cres Stria terminalis can not be resolved with current resolution L | 0.053 (0.004, 0.101) | 0.033 | 0.123 |
| Female | MD Superior longitudinal fasciculus R | 0.088 (0.019, 0.158) | 0.013 | 0.064 |
| Female | MD Superior longitudinal fasciculus L | 0.117 (0.058, 0.177) | <0.001 | 0.004 |
| Female | MD Superior fronto occipital fasciculus could be a part of anterior internal capsule R | 0.073 (0.027, 0.119) | 0.002 | 0.02 |
| Female | MD Superior fronto occipital fasciculus could be a part of anterior internal capsule L | 0.057 (0.010, 0.103) | 0.017 | 0.074 |
| Female | MD Uncinate fasciculus R | 0.077 (0.007, 0.147) | 0.031 | 0.118 |
| Female | MD Uncinate fasciculus L | -0.023 (-0.089, 0.043) | 0.498 | 0.644 |
| Female | MD Tapetum R | 0.077 (-0.002, 0.156) | 0.058 | 0.178 |
| Female | MD Tapetum L | 0.071 (-0.014, 0.156) | 0.101 | 0.28 |

Models were adjusted for age, sex, and total intracranial volume (TIV)


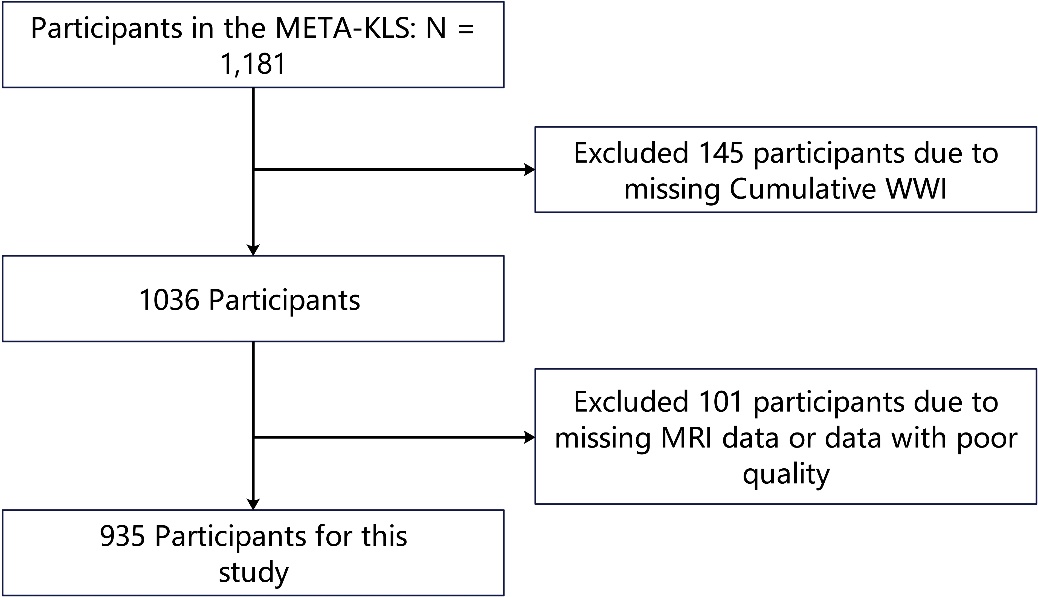


Supplementary Figure S1. Flowchart of participant selection.

META-KLS, Multimodality Medical Imaging Study Based on Kailuan Study; WWI, weight-adjusted-waist index; MRI, magnetic resonance imaging.


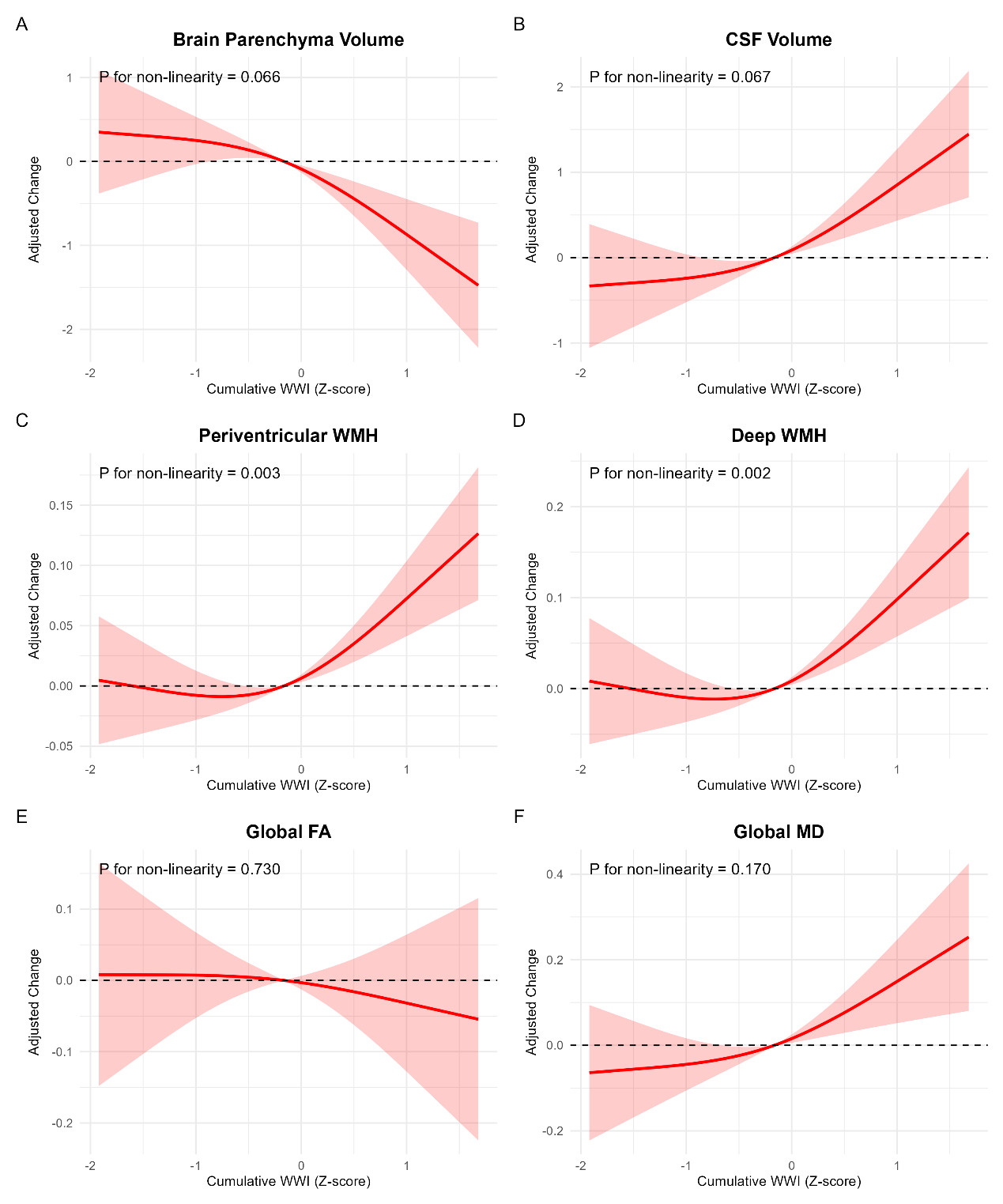


Supplementary Figure S2. Dose-response relationships between the weight-adjusted-waist index (WWI) and neuroimaging markers of brain health.

The solid curves represent the estimated Beta coefficients (β) derived from multivariable linear regression models, showcasing the association between WWI and each neuroimaging metric. The shaded areas indicate the 95% confidence intervals.


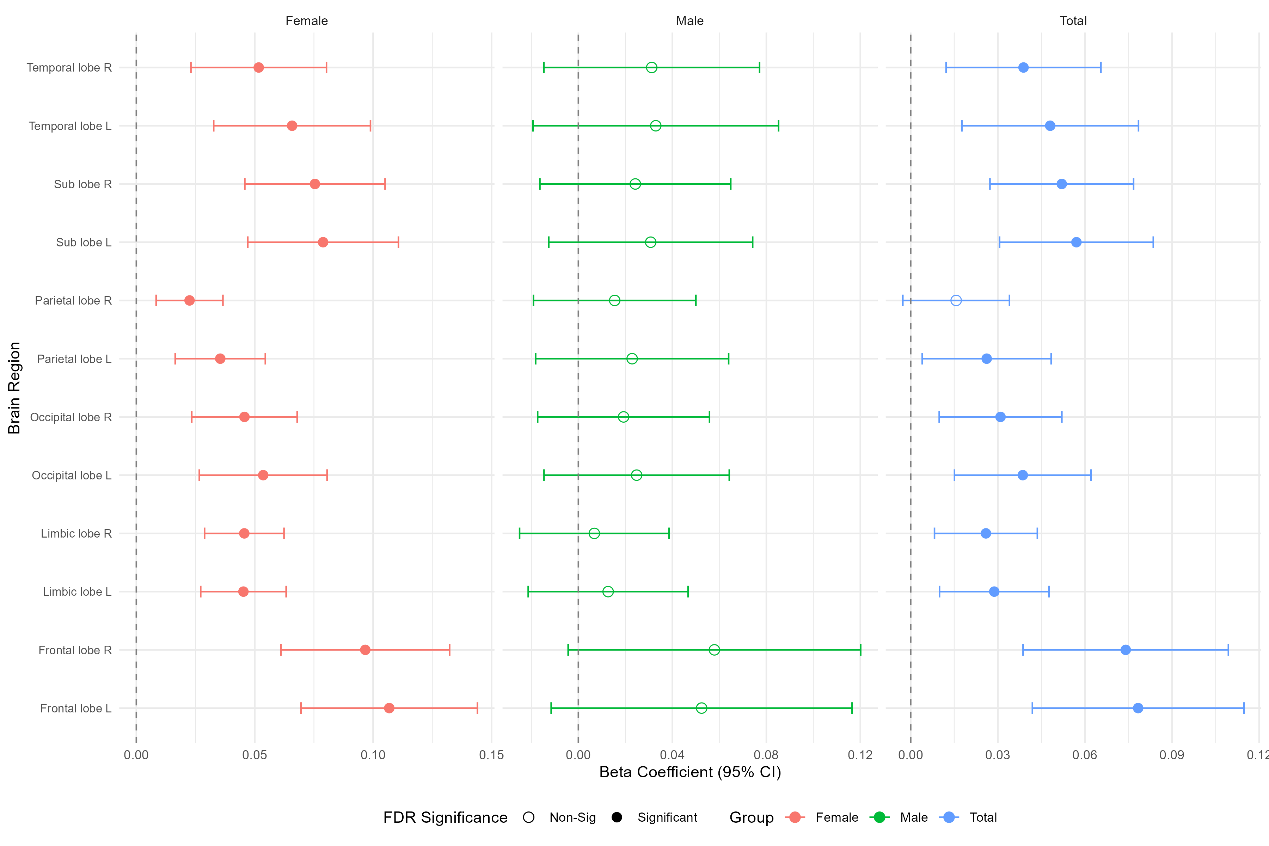


Supplementary Figure S3. Association of the weight-adjusted-waist index with regional white matter hyperintensities.

Forest plot displaying the beta coefficients (95% CI) for the association between standardized WWI and log-transformed white matter hyperintensity (WMH) volumes in lobar regions. Models were adjusted for age, sex (in the total sample), and TIV. Analyses were stratified by gender. Solid circles indicate statistical significance after FDR correction (*P* < 0.05). Abbreviations: WMH, white matter hyperintensity; TIV, total intracranial volume.


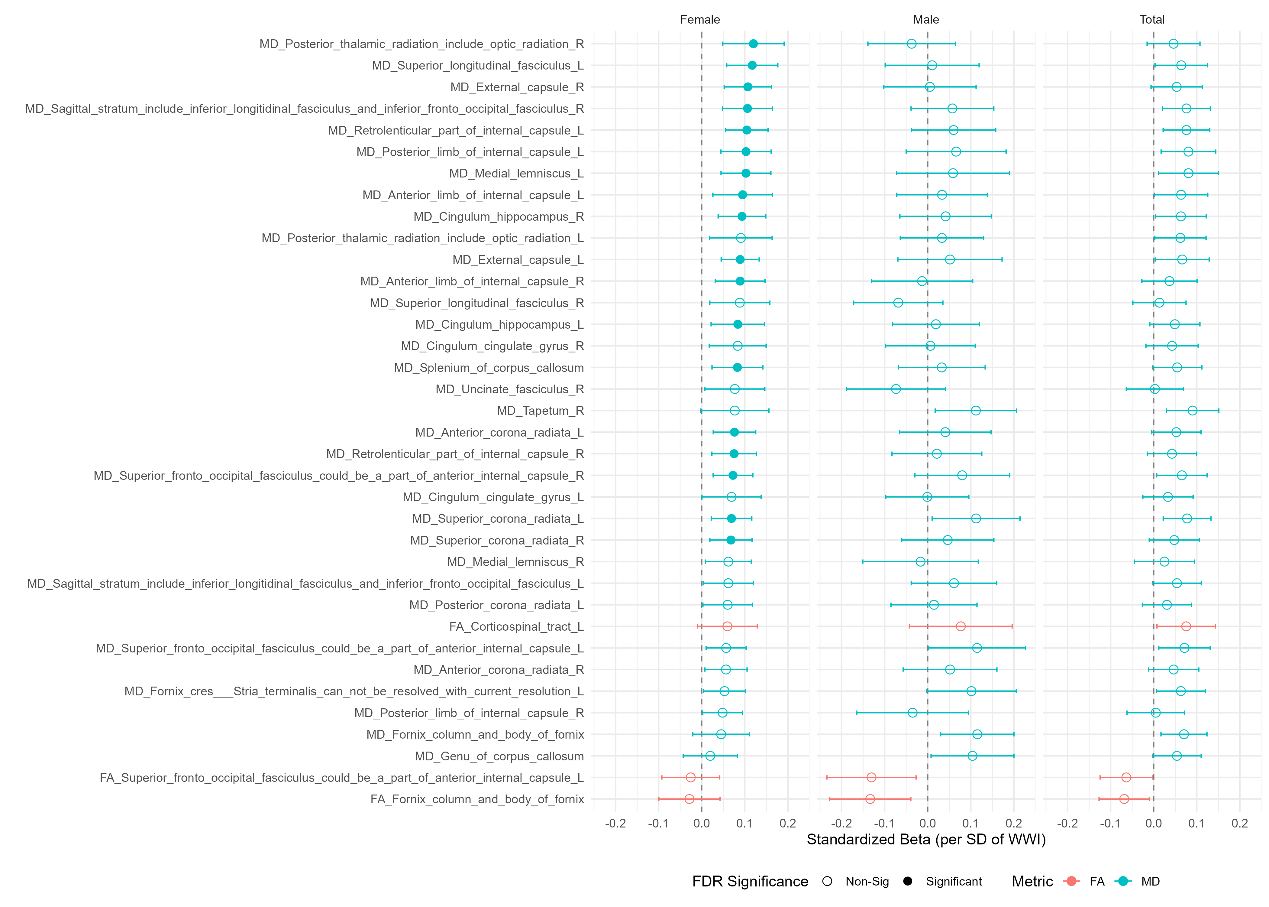


Supplementary Figure S4. Association of the weight-adjusted-waist index with diffusion tensor imaging metrics.

Forest plot illustrating the standardized beta coefficients (95% CI) for the association between standardized WWI and diffusion tensor imaging (DTI) metrics, including fractional anisotropy (FA) and mean diffusivity (MD), across various white matter tracts. Models were adjusted for age, sex (in the total sample), and TIV. Solid circles indicate FDR-corrected *P* < 0.05. Abbreviations: DTI, diffusion tensor imaging; FA, fractional anisotropy; MD, mean diffusivity.s
